# Shotgun proteomics reveals putative polyesterases in the secretome of the rock-inhabiting fungus *Knufia chersonesos*

**DOI:** 10.1038/s41598-020-66256-7

**Published:** 2020-06-17

**Authors:** Donatella Tesei, Felice Quartinello, Georg M. Guebitz, Doris Ribitsch, Katharina Nöbauer, Ebrahim Razzazi-Fazeli, Katja Sterflinger

**Affiliations:** 1 Institute of Microbiology and Microbial Biotechnology, University of Natural Resources and Life Sciences, Muthgasse 18, 1190 Vienna, Austria; 2 Institute of Environmental Biotechnology, University of Natural Resources and Life Sciences, Konrad Lorenz Strasse 20, 3430 Tulln, Austria; 3 Austrian Centre of Industrial Biotechnology, Konrad Lorenz Strasse 20, 3430 Tulln, Austria; 4 VetCore Facility for Research, University of Veterinary Medicine, Veterinärplatz 1, 1210 Vienna, Austria

**Keywords:** Biotechnology, Microbiology, Proteomics

## Abstract

*Knufia chersonesos* is an ascomycotal representative of black fungi, a morphological group of polyextremotolerant melanotic fungi, whose ability to resort to recalcitrant carbon sources makes it an interesting candidate for degradation purposes. A secretome screening towards polyesterases was carried out for the fungus and its non-melanized mutant, grown in presence of the synthetic copolyester Polybutylene adipate terephthalate (PBAT) as additional or sole carbon source, and resulted in the identification of 37 esterolytic and lipolytic enzymes across the established cultivation conditions. Quantitative proteomics allowed to unveil 9 proteins being constitutively expressed at all conditions and 7 which were instead detected as up-regulated by PBAT exposure. Protein functional analysis and structure prediction indicated similarity of these enzymes to microbial polyesterases of known biotechnological use such as MHETase from *Ideonella sakaiensis* and CalA from *Candida albicans*. For both strains, PBAT hydrolysis was recorded at all cultivation conditions and primarily the corresponding monomers were released, which suggests degradation to the polymer’s smallest building block. The work presented here aims to demonstrate how investigations of the secretome can provide new insights into the eco-physiology of polymer degrading fungi and ultimately aid the identification of novel enzymes with potential application in polymer processing, recycling and degradation.

## Introduction

Synthetic polymeric materials such as polyesters are ubiquitously present in our daily life because of their numerous applications^1,2^. The high production rate together with their resistance to environmental influences and recalcitrance to microbial attacks, has however strongly contributed to their undesirable accumulation in nature and hence to environmental and waste management issues^3^. In order to counteract the resulting economic and ecological damage^4^, over the last two decades a focus has been set on the development of more environmental friendly materials^5,6^. Consequently, aliphatic-aromatic copolyesters such as Poly (1,4-butylene adipate-co-terephthalate) (PBAT) have been established, which combine beneficial characteristics of conventional polymers with biodegradability^7^. Based on its mechanical properties similar to that of the very common polyethylene (PE), PBAT fulfils requirements for the usage as food packaging and lamination material, as organic waste bags and as agricultural equipment like mulch films^8,9^, yet while being easier to biodegrade^6,10,11^. Apart from biodegradability, enzymatic hydrolysis would enable recovery of the polymer building blocks as a novel and environmentally friendly strategy for recycling of polymers^12^.

Microbial activities against PBAT have been shown in bacteria and to a lesser extent in fungi from soil^13–17^, by esterolytic hydrolases^7,15,18,19^. Typically, the investigation of PBAT degrading enzymes involves functional screenings and protein isolation through chromatographic purification^14,16,20,21^. Alternatively, *in silico* screenings of OMICS databases are performed prior to heterologous expression of the target enzymes^12^. With only a few exceptions^15^, the majority of the studies however lack a comprehensive investigation at the proteome level of the possible effects of the polymer on induction of enzyme production by microorganisms. Degradation of polyesters is largely influenced by microbial activity in the environment and more directly by the asset of secreted hydrolytic enzymes. A proteomic screening of the secretome – alongside other techniques for the assessment of polymer hydrolysis – therefore represents a promising approach towards the detection of novel polyesterases in view of the recycling or the reuse of hybrid types of co-polyesters like PBAT, in a cost-effective manner^4^.

Most of the screenings of microorganism with PBAT degradation capacities have hitherto focused on bacteria, thereby the potential of fungal hydrolases has not yet been fully exploited. Yet fungi would deserve receiving attention; especially extremophilic and extremotolerant species, as they offer a source of proteins and compounds with functionality and stability under uttermost values of various physico-chemical parameters, in lieu of mesophilic ones^22^. Some of the most stress-tolerant fungal isolates known to date are found in the group of black fungi, a heterogeneous assembly of melanotic microfungi, whose geographical distribution includes extreme and unusual habitats^23^. In recent decades, the mechanisms of survival displayed by these organisms and their not yet fully elucidated eco-physiology have raised increasing interest, thereby the black fungi group includes nowadays a number of emerging model organisms^24–28^. Recently, the bioremediation potential of some species was recognized, along with the ability of species isolated from oligotrophic environments to resort to recalcitrant carbon sources spurned by other microorganisms^29–31^. Such a nutritional physiology, as demonstrated in the rock-associated *Knufia chersonesos*^32^, can translate into the aptitude to tolerate and grow on monoaromatic compounds and to possibly feed on other types of alternative carbon sources. The screening of this and other black fungi species might therefore hold great potential also in view of the biodegradation of synthetic polyesters.

In the present study the extremotolerant black fungus *K. chersonesos* and its non-melanised counterpart^33^ were tested for their ability to hydrolyse PBAT, with an eye to the impact of cultivation conditions and cell-wall melanisation on the strains’ degradation skills. By doing that, we show how the use of proteomics aided the detection of novel polyesterases, whose levels are modulated upon exposure to the polymer in the culture medium. Their abundance patterns, predicted functions and role in the polymer breakdown were investigated.

## Results

### **Hydrolysis of PBAT during cultivation of*K. chersonesos***

When cultivated in absence and presence of the polyester PBAT, the amount of protein secreted by *K. chersonesos* appeared to be strictly linked to the nutrient availability in the media and quite unrelated to the presence of the polymer, as also visualised by SDS-PAGE (Supplementary Figs. S1, S2a). Likewise, the recovered fungal biomass at the end of the incubation time positively reflected the media composition with lower dry weight biomass amounts detected in minimal medium (Supplementary Fig. S2).

**Figure 1:**
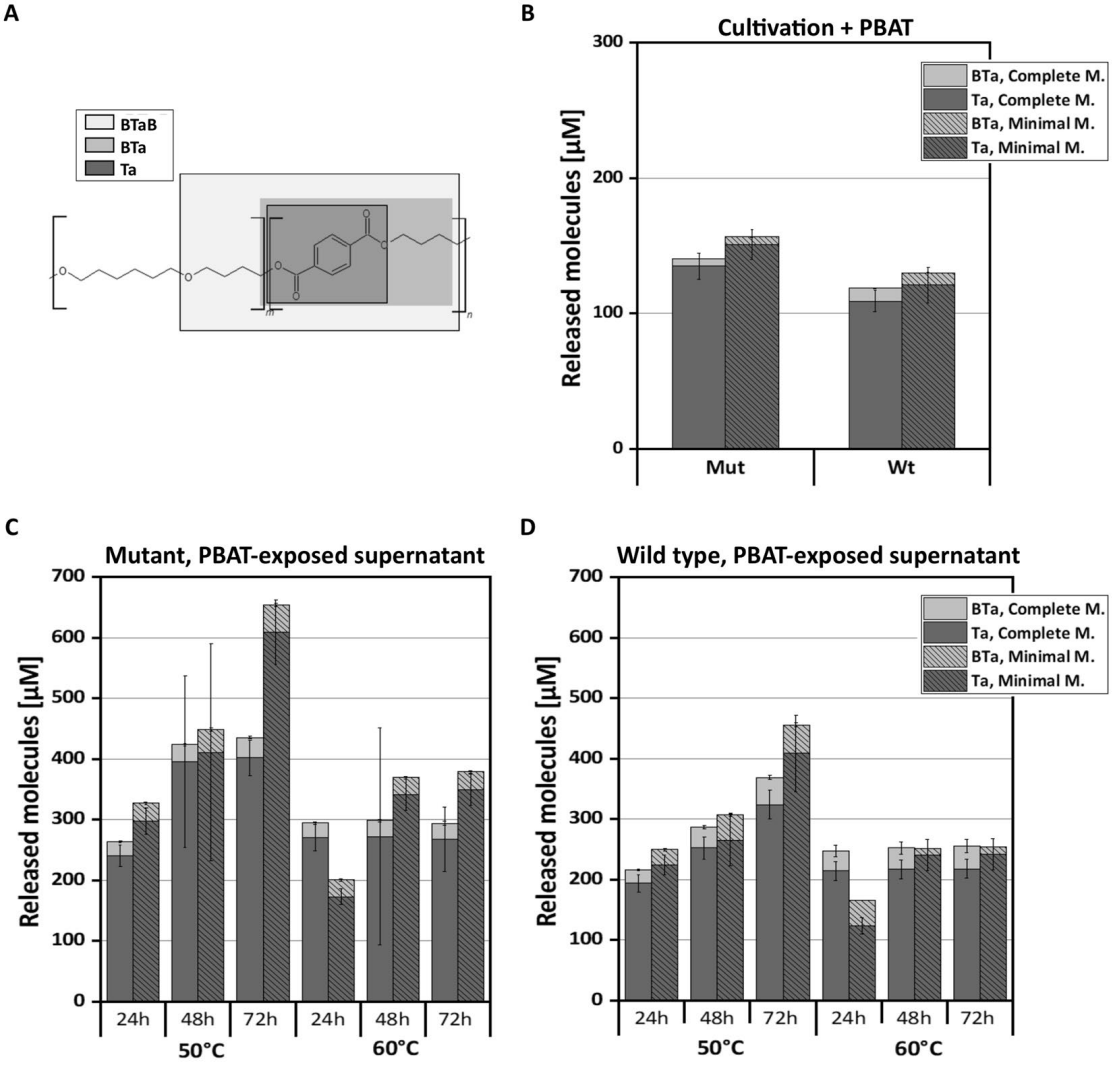
Hydrolytic activity of PBAT-exposed and unexposed culture supernatants of *K. chersonesos* MA5789 wild type (Wt) and *K. chersonesos* MA5790 mutant (Mut) from complete and minimal medium. (**A**) Chemical structure of PBAT (ACD/ChemSketch) consisting of butylene terephtalate (n) and butylene adipate (m) where the three hydrolysis products bis(4-hydroxybutyl) terephthalate (BtaB), mono(4-hydroxybutyl) terephthalate (BTa) and terephthalic acid (Ta) are displayed. (**B**) Amounts of released molecules measured in the culture supernatant after a 14 days cultivation at 21 °C in presence of PBAT and (**C,D**) after further incubation of the exposed supernatants (cell-free) with PBAT at 50 °C and 60 °C for 24, 48, and 72 h. PBAT hydrolysis products were not detected in negative controls with no fungal organisms and unexposed cultures with no PBAT. The values seen in the negative controls (not shown) thereby demonstrated that the sample acidification by HCl prior to HPLC did not lead per se to hydrolysis of the polymer. Values are means of three biological replicates and standard deviations are shown as bars. Complete M.: complete medium; Minimal M.: minimal medium. All graphs were created using Origin Pro v 9.5 (https://www.originlab.com/origin).

The ability of *K. chersonesos* secreted enzymes to hydrolyse PBAT was investigated by measuring oligomeric and monomeric hydrolysis products BTaB, BTa and Ta in the culture supernatants. The levels of the molecules detected in both the Wt and Mut strains in two different media, are shown in Fig. 1B. The exposure to PBAT resulted in the detection of prevalently Ta, and of BTa in smaller concentrations. The trimer BTaB was not detected. The amounts of Ta released by both strains cultivated in complete medium were in the same order of magnitude (i.e 134 and 109 µM in the Mut and in the Wt, respectively). A higher concentration of Ta was detected in minimal medium, where it reached values up to 150 µM in the Mut and up to 120 µM in the Wt. None of the PBAT hydrolysis products were detected in the unexposed cultures (no polymer added) and in the negative control cultures (medium and polymer, no fungus).

Upon incubation of both *K. chersonesos* strains in either complete or minimal medium solely with Ta to investigate the fungus aptitude to use the released product as possible carbon source, no significant change in the detected Ta concentration (i.e. 965 μM in both control and treatment cultures at timepoint 0 and after 2 weekswas seen). Hence, Ta detected after cultivation of *K. chersonesos* in the presence of PBAT can be related to extracellular enzymatic hydrolysis.

### **Hydrolysis of PBAT by cell-free supernatants of*K. chersonesos***

To evaluate temperature-related changes in the hydrolytic activity, the cell-free PBAT-exposed supernatants were incubated with PBAT at temperatures higher than the cultivation temperature (i.e. 21 °C). Higher amounts of monomer (Ta) and dimer (BTa) were observed following incubation at 50 and 60 °C (Fig. 1C,D) in comparison to 21 °C (Fig. 1B) while the trimer (BTaB) was not detected in any of the experimental sets. Moreover, higher amounts of released molecules were measured during incubation in minimal medium for the two strains. At 50 °C, both Mut and Wt exhibited a degradation profile that increased over time with 609 and 408 µM of Ta representing the uppermost values detected in minimal medium, respectively. At 60 °C no increase in concentration was observed after 48 h of incubation, possibly due to the instability and/or denaturation of the putative polyesterases^7^. Variability was observed among the biological replicates of the 48 h samples from the Mut at both temperatures, most likely because of interfering molecules in the supernatant. The precipitation of substrate or entrapment into the fungal biomass, could also be speculated in this case.

### **Proteome screening of unexposed and PBAT-exposed supernatants**

**Figure 2:**
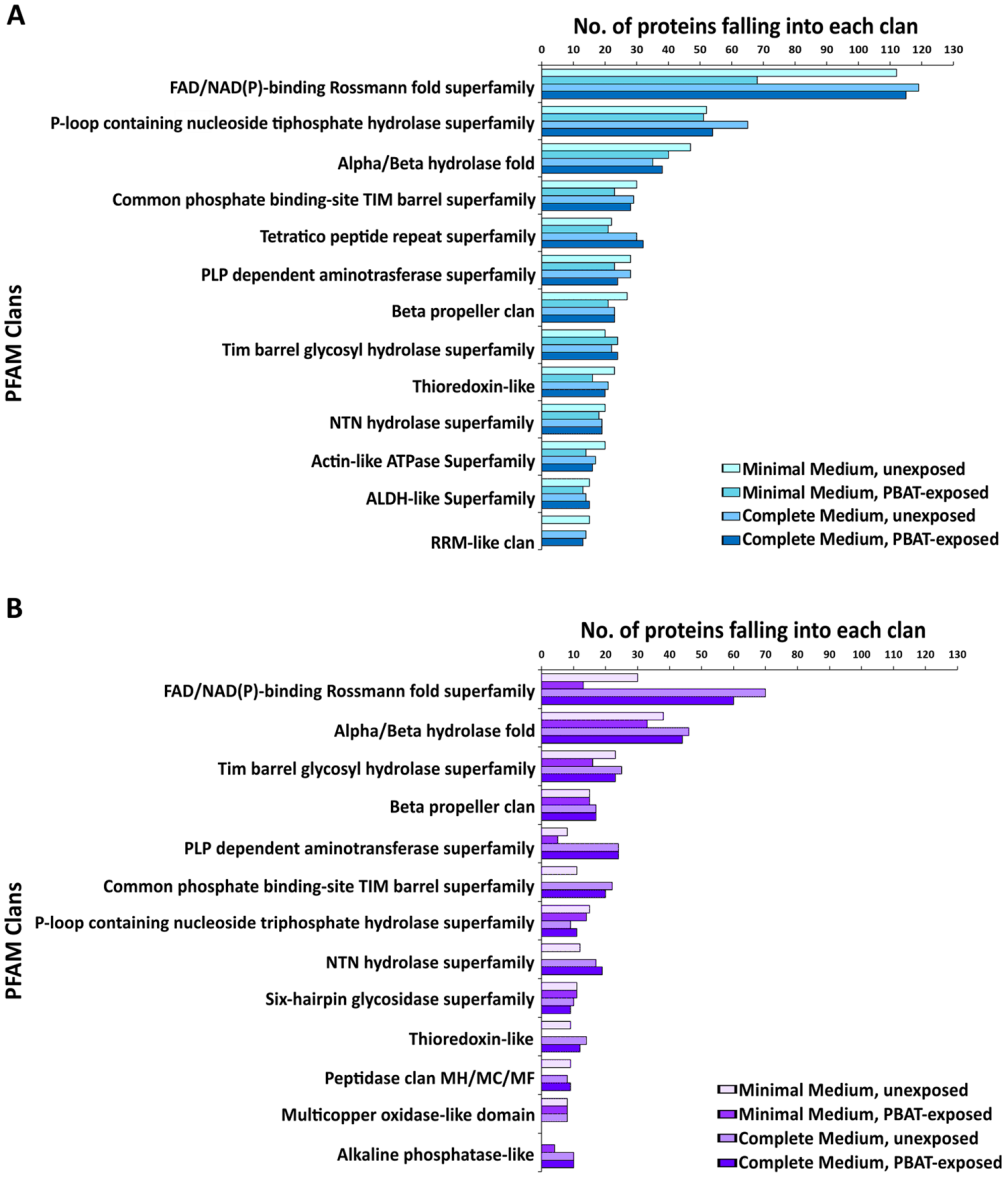
Distribution of the PFAM clans with the highest number of associated proteins among the 8 different experimental conditions in *K. chersonesos* Wt (**A**) and Mut (**B**). The graphs were created using Origin Pro v 9.5 (https://www.originlab.com/origin).

To identify PBAT-hydrolytic enzymes and their relative abundance under different cultivation conditions, exposed and unexposed supernatants were subjected to proteomic analyses. Across the 8 conditions established at 21 °C and their biological replicates, a total of 1848 proteins were detected by MS/MS and 1730 were identified by homology search. In line with the recorded protein concentrations, the number of identified proteins was higher in complete than in minimal medium in both strains. Moreover, twice the number of proteins were identified in *K. chersonesos* Wt in comparison to the mutant (Supplementary Table S1). An overview of the biological functions of the secreted proteins was obtained through a PFAM analysis. Distribution of the PFAM clans with the highest number of associated proteins among all the experimental conditions is displayed in Fig. 2. Proteins from 241 PFAM clans were detected. Some of the clans were common to Wt and Mut, others were instead unique to each strain. CL0063 (FAD/NAD(P)-binding Rossmann-fold superfamily), including redox proteins, was the most represented clan in both fungal strains. Following CL0063, clans encompassing proteins with hydrolytic activity were found. The second most abundant protein group in the Wt, CL0023 (P-loop containing nucleoside triphosphate hydrolase superfamily), contained AAA family proteins with chaperone-like functions. Clan CL0028 was second per abundance in the mutant strain and third in the Wt. The clan includes alpha/beta hydrolase fold enzymes with widely differing catalytic functions but all characterized by a Nucleophile-His-Acid catalytic triad, which is substrate-specific. Members in this family include lipases, esterases, serine carboxypeptidases, lyases and others. The third largest clan detected in the mutant was CL0058, which represents a range of glycosyl hydrolase enzymes possessing a TIM barrel fold, a widespread group of enzymes with multiple roles in the cell basic energy metabolism. Despite the discrepancy in the total number of different proteins detected in the two strains (i.e. 1791 in the Wt and 1070 in the Mut), the count of hydrolases resulted to be very comparable also amid different experimental conditions.

Insights into the functions of the secretome were integrated with data about protein biological processes. GO-Slim enrichment analysis identified a total of 287 hydrolases, 37 of which were predicted to have esterase, lipase and cutinase activity. These classes of enzymes were chosen based on their reported hydrolytic activity towards polymers^18^. Around 51% of these proteins were predicted to be secreted, the rest were intracellular proteins detected outside the cell possibly due to mechanical stress and cell death^34^. The protein identities were confirmed through search for characterized homologues within the UniProtKB database. As shown in Supplementary Table S2, the maximum rate of sequence similarity was often found with proteins belonging to the black yeast *E. dermatitidis* whose genome was used in the present study as training database. The list of closely related organisms included mainly species belonging to the order *Chaetothyriales* – which the genus *Knufia* belongs to – and *Capnodiales*. The identity values associated to the homologous proteins were overall higher than 50% except for 7 proteins, due to lack of sequence similarity in UniProtKb. Nevertheless, a comparison of these IDs with those obtained through searches in PFAM, revealed that the results of homology searches in the two databases were consistent (Table 1).

**Table 1:** List of hydrolases with predicted esterase, lipase and cutinase activity, identified in culture supernatants of *K. chersonesos* Wt (WT), *K. chersonesos* Mut (MUT) or in both (X) across the cultivation conditions established at 21 °C. The hyphen (—) indicates that the protein was not detected.

Protein accession number ^a^	UniProtKb Name	HMMER accession number	HMMER Name	PFAM Clan	Minimal Medium		Complete Medium	
					Unexposed	Exposed	Unexposed	Exposed
**g1109.t1**	**Secretory lipase**	**PF03583.13**	**LIP**	**CL0028**	**X**	**X**	**X**	**X**
**g1329.t1**	**Carboxylic ester hydrolase**	**PF00135.27**	**Coesterase**	**CL0028**	**X**	**X**	**X**	**X**
**g1587.t1**	Triacylglycerol lipase	PF01764.24	Lipase_3	CL0028	—	—	MUT	MUT
**g2066.t1**	Esterase LovG	PF03959.12	FSH1	CL0028	—	—	MUT	MUT
**g2279.t1**	**Carboxylic ester hydrolase**	**PF00135.27**	**Coesterase**	**CL0028**	**X**	**X**	**X**	**X**
**g2917.t1**	**Carbohydrate esterase family 1 protein**	**n/a**	**no matches**	**n/a**	**X**	**X**	**X**	**X**
**g2930.t1**	Carboxymethylenebutenolidase	PF01738.17	DLH	CL0028	X	—	X	X
**g3032.t1**	Phospholipase	PF13091.6	PF13091.6	CL0479	MUT	WT	WT	WT
**g3128.t1**	Protein ssh4	PF02112.15	PDEase_II	CL0381	—	—	MUT	MUT
**g3531.t1**	S-formylglutathione hydrolase	PF00756.19	Esterase	CL0028	WT	WT	X	X
**g3802.t1**	Triacylglycerol lipase	PF01764.24	Lipase_3	CL0028	X	MUT	MUT	—
**g3878.t1**	Thioesterase family protein	PF03061.21	4HBT	CL0050	—	—	MUT	MUT
**g4102.t1**	Isoamyl acetate-hydrolysing esterase 1 like protein	PF13472.5	Lipase_GDSL_2	CL0264	—	—	MUT	MUT
**g4295.t1**	Cutinase	PF01083.21	Cutinase	CL0028	—	X	MUT	-
**g4612.t1**	**Carboxylic ester hydrolase**	**PF00135.27**	**Coesterase**	**CL0028**	**X**	**X**	**X**	**X**
**g4621.t1**	Cutinase	PF01083.21	Cutinase	CL0028	WT	—	—	—
**g5276.t1**	Ubiquitin thiolesterase	PF00443.28	UCH	CL0125	—	—	WT	WT
**g5383.t1**	Carboxylic ester hydrolase	PF00135.27	Coesterase	CL0028	X	X	MUT	X
**g5594.t1**	Triacylglycerol lipase	PF07859.13	Abhydrolase_3	CL0028	X	—	X	X
**g5645.t1**	Carboxylic ester hydrolase	PF00135.27	Coesterase	CL0028	—	—	MUT	MUT
**g5761.t1**	Carboxylic ester hydrolase	PF00135.27	Coesterase	CL0028	X	X	MUT	X
**g5776.t1**	Putative esterase C31F10.02	PF03061.21	4HBT	CL0050	—	—	MUT	X
**g6131.t1**	Esterase/lipase	PF01738.17	DLH	CL0028	X	WT	MUT	X
**g6247.t1**	Lysophospholipase	PF01735.17	PLA2_B	CL0323	X	X	MUT	X
**g6560.t1**	Carboxylesterase	PF07859.13	Abhydrolase_3	CL0028	WT	—	MUT	X
**g6652.t1**	Carboxylic ester hydrolase	PF00135.27	Coesterase	CL0028	X	X	X	WT
**g723.t1**	Carboxylic ester hydrolase	PF00135.27	Coesterase	CL0028	X	X	MUT	X
**g7247.t1**	**PI-PLC X domain-containing protein**	**n/a**	**no matches**	**n/a**	**X**	**X**	**X**	**X**
**g7566.t1**	Carboxylic ester hydrolase	PF07519.10	Tannase	CL0028	X	X	MUT	X
**g7567.t1**	Para-nitrobenzyl esterase	PF00135.27	Coesterase	CL0028	X	X	X	—
**g7569.t1**	**Cutinase**	**PF01083.21**	**Cutinase**	**CL0028**	**X**	**X**	**X**	**X**
**g762.t1**	Putative erythromycin esterase	PF05139.13	Erythro_esterase	CL0572	—	—	WT	WT
**g7983.t1**	**Carboxylic ester hydrolase**	**PF00135.27**	**Coesterase**	**CL0028**	**X**	**X**	**X**	**X**
**g8915.t1**	Carboxylic ester hydrolase	PF00135.27	COesterase	CL0028	—	MUT	—	—
**g8978.t1**	**Carboxylic ester hydrolase**	**PF00135.27**	**Coesterase**	**CL0028**	**X**	**X**	**X**	**X**
**g9204.t1**	Coesterase	PF00135.27	COesterase	CL0028	MUT	X	—	—
**g9456.t1**	Putative esterase C31F10.02	PF03061.21	4HBT	CL0050	X	WT	X	X

Proteins expressed at all cultivation conditions and in both strains are highlighted in bold.

^a^Protein accession number in the *K. chersonesos* database of ab initio translated proteins.

Distribution of the polyesterases among diverse cultivation conditions is summarized in Table 1: around 75% were identified at minimal medium, while 91% were detected in complete medium. Out of the 37 enzymes, 9 were found at all cultivation conditions in both strains, 3 only in the Wt and 7 exclusively in the mutant. As revealed by the MS-based quantitative analysis, the abundance levels of these and of a number of other proteins were influenced by exposure to PBAT.

### **Overview of secretome quantitative analysis**

To get an insight into significant re-arrangements of the secretome when exposed to PBAT, a quantitative analysis was carried out by comparing experimental sets. The clustering of secretomes, biological and technical replicates included, was confirmed by PCA and HC analysis (Supplementary Figs. S3 and S4). The number of proteins exhibiting significant modulation (*p*-value ≤ 0.05, fold change ≥2) is displayed in Table 2. Protein regulation was detected at each experimental set, however PBAT-dependent changes in the abundance of polyesterases could be observed in the secretomes of *K. chersonesos* Wt and Mut only when grown in minimal medium. The results of whole secretome protein differential abundance analysis (minimal medium, unexposed v/s PBAT-exposed), including overrepresented biological processes GO terms, are discussed in detail in Supplementary Information Section 1.4.

**Table 2:** Number of proteins detected as significantly regulated at each experimental set.

Strain	Experimental set	Total No. regulated proteins	No. regulated hydrolases	No. down-regulated proteins (hydrolases)	No. up-regulated proteins (hydrolases)
*K. chersonesos* Wt	Complete medium, Unexposed v/s PBAT-exposed	55	7	17 **(2)**	38 **(5)**
	Minimal medium, Unexposed v/s PBAT-exposed	122	23	55 **(5)**	67 **(18)**
	Unexposed, Minimal medium v/s Complete medium	134	29	25 **(2)**	109 **(27)**
	PBAT-exposed, Minimal medium v/s Complete medium	127	28	49 **(5)**	78 **(23)**
*K. chersonesos* Mut	Complete medium, Unexposed v/s PBAT-exposed	48	7	26 **(4)**	22 **(3)**
	Minimal medium, Unexposed v/s PBAT-exposed	169	17	142 **(16)**	27 **(1)**
	Unexposed, Minimal medium v/s Complete medium	131	19	86 **(13)**	45 **(6)**
	PBAT-exposed, Minimal medium v/s Complete medium	194	28	153 **(25)**	41 **(3)**

### **Differential abundance and characterization of PBAT-regulated polyesterases**

**Figure 3:**
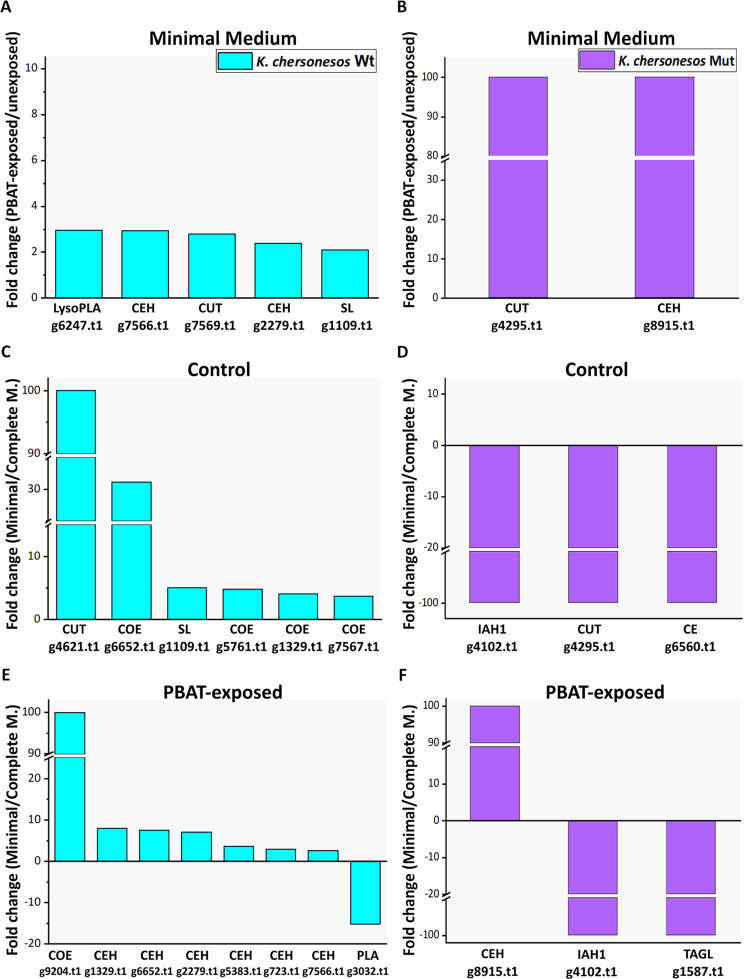
Classes of secreted hydrolytic enzymes with predicted esterase, lipase and cutinase activity detected as regulated in the culture supernatant of *K. chersonesos* Wt (**A,C,E**) and *K. chersonesos* Mut (**B,D,F**). (**A,B**) Experimental set: PBAT-exposed v/s unexposed secretomes. Up- regulated enzymes under PBAT exposure at minimal medium condition, are shown; (**C–F**) Experimental set: minimal medium v/s complete medium. Up- and down-regulated enzymes at minimal medium condition in unexposed (**C,D**) and PBAT-exposed supernatants (**E,F**), are shown. No significant regulation of target enzymes was detected in complete medium in both Wt and Mut (data not shown). Protein abundances are expressed as fold change. Proteins were evaluated for increased or decreased abundance using a cut-off value of fold change of ≥2. Fold change equal to 100 or −100 indicates proteins exclusively found in one out of the two experimental conditions (i.e. On/Off proteins). LysoPLA: Lysophospholipase; CEH: Carboxylic ester hydrolase; CUT: cutinase; SL: secretory lipase; COE: COesterase; PLA: Phospholipase; TAGL: triacylglycerol lipase; IAH1: Isoamyl acetate hydrolysing esterase 1. Minimal: minimal medium; Complete: Complete Medium. The graphs were generated by Origin Pro v 9.5 (https://www.originlab.com/origin).

The quantitative analysis of PBAT-exposed v/s unexposed secretomes of *K. chersonesos* Wt and Mut from minimal medium resulted in the identification of 7 polyesterases regulated in presence of PBAT (Fig. 3A,B). All 5 enzymes found in the Wt showed a nearly 3-fold up-regulation. The 2 enzymes found in the mutant instead, were solely detected in the PBAT-exposed secretome (i.e. 44295t1 and g8915.t1). Bioinformatics analysis supported a more in deep characterization of these proteins.

Protein g7566.t1/A0A1Y2EA24_9PEZI and g2279.t1/A0A1J9RJA8_9PEZI were detected as homologous of extracellular carboxylic ester hydrolases (CEHs) with no further characterization to the protein class level being possible on UniProtKb. Computation of pI and Mw resulted in 4.24/63217.74 and 4.67/58682.56 for g7566.t1 and g2279.t1, respectively. Protein g6247.t1 and g1109.t1 were both identified as lipases, i.e. lysophospholipase/A0A0D2AQ38_9EURO and secretory lipase/A0A2K3Q6V2_9HYPO, respectively. The first (pI/Mw of 4.31/70943.42) was predicted as an anchored component of the plasma membrane based on the presence of a GPI-anchor, while the lipase (pI/Mw of 4.65/52508.87) was deemed as extracellular (Supplementary Table S3). Cutinase g7569.t1/A0A0D2AG04_9PEZI was predicted to be a secreted enzyme. Theoretical pI/Mw were 4.39/26398.30, the latter slightly above the usual Mw range of fungal cutinases (i.e. 20–25 kDa^35^). Cutinase g4295.t1/W2RQJ3_9EURO and CEH g8915.t1/A0A1L7WTP0_9HELO were exclusively detected in *K. chersonesos* Mut induced secretome, this suggesting their nature of On/Off proteins (Fig. 3B; Supplementary Table S4). Whereas the cutinase (pI/Mw: 4.94/22355.23) undergoes secretion into the extracellular space, the CEH (pI/Mw: 4.20 /57588.75) was predicted as an anchored component of plasma membrane.

3-D structure prediction using modelling by Phyre2 (Table 3) was additionally carried out for the PBAT-regulated enzymes. All 7 proteins exhibited Phyre2 confidence score of 100. Protein g7566.t1 displayed the highest alignment coverage with the feruloyl esterase B from *Aspergillus oryzae*^36^ (AoFaeB), while showing high structural similarity also to the *Ideonella sakaiensis* mono(2-hydroxyethyl) terephthalate hydrolase (MHETase). AoFaeB is known for its ability to hydrolyse the plant cell wall and to be involved in the synthesis of several bioactive compounds^37^. MHETase, which is reminiscent of tannases and feruloyl esterases, was recently reported to be involved in a two-step degradation of PET film together with PETase^38,39^. Both proteins possess a classic α/β hydrolase domain and a lid domain conferring substrate specificity^36,38^. Protein g2279.t1 displayed the highest similarity with an alpha-esterase-7-carboxylesterase^40^. The lysophospholipase (g6247.t1) resembled the human cytosolic phospholipase A2. The secretory lipase g1109.t1 matched the template lipase A from *Candida antarctica* (CalA) known for its ability to maintain the hydrolytic activity in organic solvents at high temperatures and over a broad pH range. CalA is reported to operate via the common mechanisms of serine hydrolases. Furthermore, it exhibits a preference for long-chained carboxylic acids and for the more extended trans-isomers of fatty acids, where most other lipases favour cis-fatty acids^41^. Both the cutinases had proteins from *Trichoderma reesei* as template. While g7569.t1 matched a cutinase-like protein (i.e. acetylxylan esterase) with a role in the hydrolysis of xylan from hardwood^42^, protein g4295.t1 resembled *Tr* cutinase. In contrast with classical cutinases, *Tr* cutinase is reported to have kinetic and structural features of true lipases, including a lid-covered active site, as well as to have optimal activity at acidic pH and specificity for long-chain triglycerides^43^. Protein g8915.t1 matched instead a cholinesterase (i.e. butyrylcholinesterase), specialized family of enzymes that hydrolyse choline-based esters, such as the neurotransmitters acetylcholine. The physiological role of choline esters-hydrolysing enzymes in fungi needs to be explored further^44^.

**Table 3:** Phyre2, 3-D structures predictions of the PBAT-regulated and of the constitutively expressed polyesterases detected in the secretome of *K. chersonesos* Wt and *K. chersonesos* Mut.

*Protein* accession No.^a^	UniProtKb accession No.	UniProtKb Protein name	Coverage %	Confidence	% i.d.	Template	Template Information
***Knufia chersonesos Wt****,* **minimal medium, PBAT-exposed**							
g6247.t1	A0A0D2AQ38_9EURO	Lysophospholipase	80	100	24	*d1cjya2*	Fold: FabD/lysophospholipase-like. Superfamily: FabD/lysophospholipase-like Family: Lysophospholipase
g1109.t1*	A0A2K3Q6V2_9HYPO	Secretory lipase	78	100	20	*c2veoA_*	PDB header: hydrolase. Chain: A: PDB Molecule: lipase a; PDB Title: x-ray structure of *Candida antarctica* lipase a in its closed state.
g2279.t1*	A0A1J9RJA8_9PEZI	Carboxylic ester hydrolase	98	100	24	*c4fg5B_*	PDB header: hydrolase. Chain: B: PDB Molecule: e3 alpha-esterase-7 carboxylesterase; PDB Title: crystal structure of the alpha-esterase-7 carboxylesterase, e3, from2 *Lucilia cuprina*
g7566.t1	A0A1Y2EA24_9PEZI	Carboxylic ester hydrolase	92	100	27	*c3wmtA_*	PDB header: hydrolase. Chain: A PDB Molecule: feruloyl esterase b-1; PDB Title: crystal structure of feruloyl esterase b from *Aspergillus oryzae*^36^
g7569.t1*	A0A0D2AG04_9PEZI	Cutinase	84	100	32	*d1qoza_*	Fold: alpha/beta-hydrolase. Superfamily: alpha/beta-hydrolase Family: cutinase-like
***Knufia chersonesos Mut*, minimal medium, PBAT-exposed**							
g4295.t1	W2RQJ3_9EURO	Cutinase	80	100	42	*c4psdA_*	PDB header: hydrolase. Chain: A: PDB Molecule: carbohydrate esterase family 5; PDB Title: structure of *Trichoderma reesei* native form.
g8915.t1	A0A1L7WTP0_9HELO	Carboxylic ester hydrolase	89	100	31	*c6i2tC_*	PDB header: hydrolase. Chain: C: PDB Molecule: cholinesterase; PDB Title: CryoEM reconstruction of full-length, fully-glycosylated human2 butyrylcholinesterase tetramer.
**Proteins expressed at all cultivation conditions, in both strains**							
g1329.t1	A0A0D2C9P1_9EURO	Carboxylic ester hydrolase (*Exophiala xenobiotica*)	93	100	29	*c4bdtA_*	PDB header: hydrolase. Chain: A: PDB Molecule:acetylcholinesterase; PDB Title: human acetylcholinesterase in complex with huprine w and fasciculin 2.
g2917.t1	M3AEN1_PSEFD	Carbohydrate esterase family 1 protein (*Pseudocercospora fijiensis, CIRAD86)*	86	100	18	*c3wlaA_*	PDB header: hydrolase. Chain: A: PDB Molecule: oxidized polyvinyl alcohol hydrolase; PDBTitle: crystal structure of *Sphingopyxis sp*. native form.
g4612.t1	A0A1L7WLC8_9HELO	Carboxylic ester hydrolase (*Phialocephala subalpina*)	95	100	28	*c4bdtA_*	PDB header: hydrolase. Chain: A: PDB Molecule:acetylcholinesterase; PDB Title: human acetylcholinesterase in complex with huprine w and fasciculin 2.
g7247.t1	A0A0N1HQ42_9EURO	PI-PLC X domain-containing protein (*Phialophora attae*)	71	100	17	*c3h4wA_*	PDB header: hydrolase. Chain: A: PDB Molecule: phosphatidylinositol-specific phospholipase c1; PDBTitle: structure of a ca+2 dependent phosphatidylinositol-specific2 phospholipase c (pi-plc) enzyme from *Streptomyces antibioticus*.
g7983.t1	A0A1L9SZB3_9EURO	Carboxylic ester hydrolase (*Aspergillus sydowii* CBS 593.65)	98	100	27	*c4fg5B_*	PDB header: hydrolase. Chain: B: PDB Molecule:e3 alpha-esterase-7 carboxylesterase; PDBTitle: crystal structure of the alpha-esterase-7 carboxylesterase, e3, from2 *Lucilia cuprina*.
g8978.t1	A0A1Q8RG26_9PEZI	Carboxylic ester hydrolase (*Colletotrichum chlorophyti*)	92	100	28	*c4bdtA_*	PDB header: hydrolase. Chain: A: PDB Molecule:acetylcholinesterase; PDB Title: human acetylcholinesterase in complex with huprine w and fasciculin 2.

^**a**^Protein accession number in the *K. chersonesos* database of ab initio translated proteins.

^*^Proteins expressed at all established cultivation conditions and up-regulated in *K. chersonesos* Wt in minimal medium upon exposure to PBAT.

### **Modelling of the carboxylic ester hydrolase g7566.t1**

**Figure 4:**
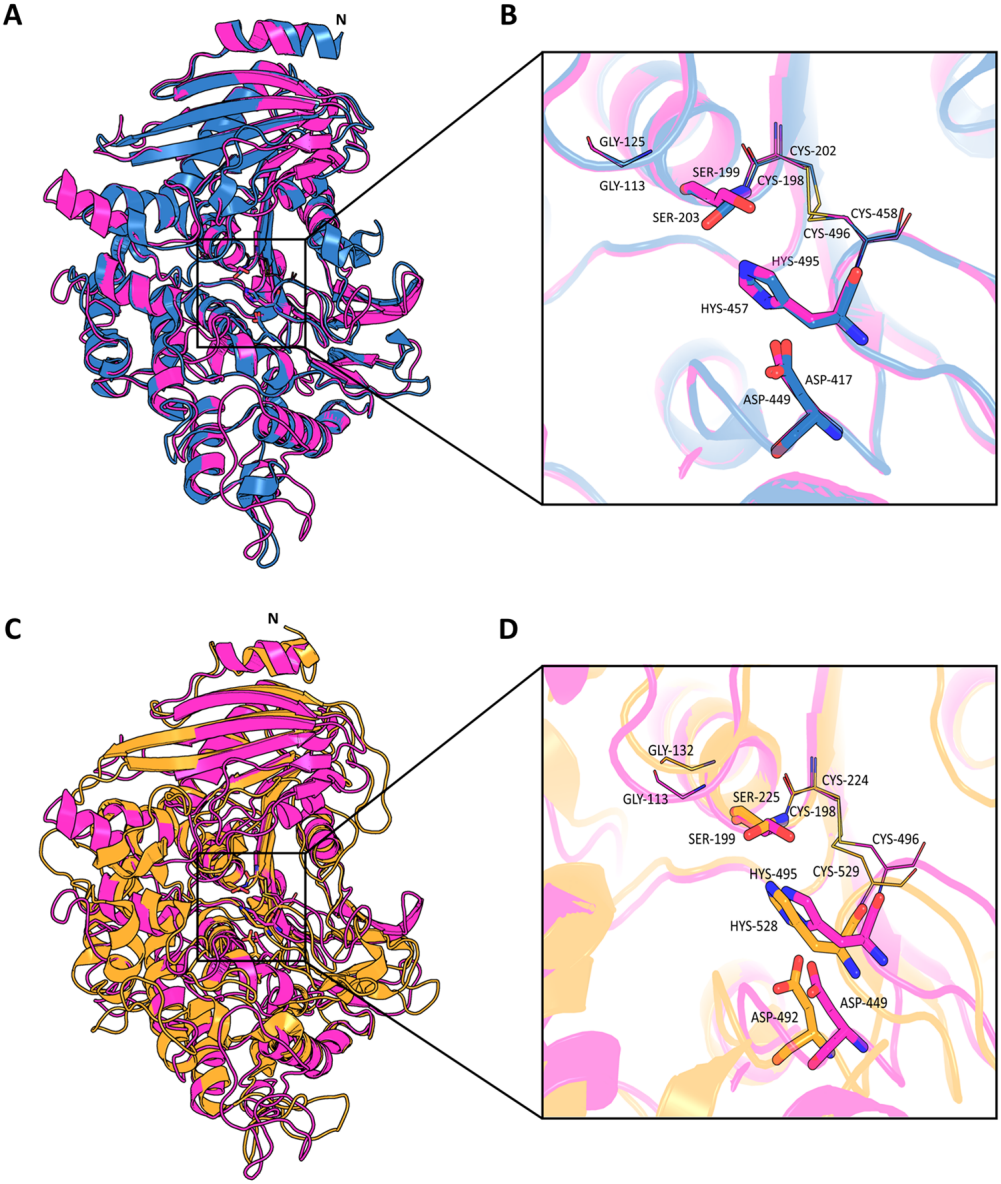
Representation of the g7566.t1 model superimposed on the crystal structure of AoFaeB (PDB: 3WMT) from *Aspergillus oryzae* (**A**) and of MHETase (PDB: 6QGB) from *Ideonella sakaiensis* (**C**), as obtained using Pymol v1.3. The active site of all three proteins is displayed (**B–D**). The sticks mode representation was used for the amino acids of the triad while Glycine and the two Cysteine of the oxyanion hole – whose disulphide bond holds Ser and His together – are represented in lines. In g7566.t1, the predicted catalytic triad consists of Ser199, His495 and Asp449 while the oxyanion hole encompasses Gly113 Cys198 and Cys496. In AoFaeB and in MHETase the amino acids involved are Ser203, His457 and Asp417 and Gly125 and Cys202-Cys458; Ser225, His528 and Asp492 and Gly132 and Cys224-Cys529, respectively. All Figures were elaborated with PyMOL Molecular Graphics System (v. 1.3, Schrödinger, LLC https://pymol.org/2/) and combined using Corel DRAW Graphics Suite 2019 (https://www.coreldraw.com/de/).

Protein g7566.t1 – upregulated in the wild type secretome in response to PBAT – shared structural similarity with the *A. oryzae* AoFaeB^36^ (92% alignment coverage, 27% sequence identity). High alignment coverage was also observed with MHETase from *I. sakaiensis*^38^ (90% alignment coverage, 23% sequence identity). Both AoFaeB (PDB: 3WMT) and MHETase crystal structure (PDB: 6QGB) were used as template to generate a 3-D model of g7566.t1. As shown in Fig. 4, the overall enzymatic structures of AoFaeB (522 aa), MHETase (596 aa) and g7566.t1 (564 aa) are structurally similar (1.50 Å RMSD and 482 residues aligned with AoFaeB and 2.20 Å RMSD and 490 with MHETase) despite a relatively low number of amino acids identities. Based on the position of the amino acids involved in the catalytic triad (Ser203, His457 and Asp417; Ser225, His528 and Asp492) and in the oxyanion hole (Gly125 and Cys202-Cys458; Gly132 and Cys224-Cys529) of AoFaeB and MHETase, respectively, residues Ser199, His495 and Asp449 and Gly113 and Cys198-Cys496 could be detected in g7566.t1. The detail of the alignment of the residues involved in the catalytic site is displayed in Fig. 4B,D, where g7566.t1 (pink), AoFaeB (blue) and MHETase (orange) are shown. MHETase was reported to exclusively hydrolyse MHET, indicating a restricted substrate specificity^39^ thus, the involvement of the CEH g7566.t1 in the hydrolysis of PBAT might be due to differences in the amino acidic sequence. This is consistent with structural observations on MHETase, which described the lid domain as the major difference to the closely related tannase and feruloyl esterases^38^. On the other hand, the only difference between MHET and the PBAT hydrolysis product BTa (this study) lies in the chain length of the alcohol (ethyl- versus butyl-). Hence, it is likely that BTa is also hydrolysed by g7566.t1, which is supported by the fact that primarily monomeric hydrolysis products from PBAT (i.e. Ta) were detected in this study.

### **Effects of the cultivation conditions on polyesterases’ differential abundance**

Quantitative proteomics analyses were performed to also investigate the influence of the culture medium in the regulation of polyesterases. The enzymes with changed abundance are displayed in Fig. 3C–F. A total of 6 proteins were over 2-fold up-regulated in the wild type unexposed secretomes from minimal medium, relative to the complete medium (Fig. 3C). Two of these proteins, CEH g6652.t1/A0A0K0KDL6_9PEZI and g1329.t1/A0A0D2C9P1_9EURO were up-regulated in minimal medium in both PBAT-exposed and unexposed secretomes. Protein up-regulation in complete medium was only detected for the phospholipase g3032.t1 (A0A0D2FB23_9EURO; Fig. 3E). In *K*. *chersonesos* Mut, out of 5 proteins, only the CEH g8915.t1/A0A1L7WTP0_9HELO was upregulated and solely detected in minimal medium secretomes exposed to PBAT. The remaining regulated proteins were exclusively found in complete medium (i.e. On/Off proteins; Fig. 3D,F). The results therefore suggest that in the mutant strain cultivation in complete medium appears to be essential not only to achieve increased abundance but also for expression of the putative polyesterases. Conversely, in the wild type, the up-regulation of the protein classes of interest is mostly triggered by suboptimal conditions of growth.

### **Constitutively expressed and strain-specific polyesterases**

Out of the 37 enzymes with predicted esterase, lipase and cutinase activity, 9 were detected at all the 8 established cultivation conditions. These enzymes (depicted in bold in Table 1) were constitutively expressed in both the wild type and the mutant regardless of PBAT’s presence and of the nutrient availability in the culture medium. Solely protein g1109.t1/secretory lipase, g2279.t1/CEH and g7569.t1/cutinase, additionally did undergo up-regulation in the wild type in minimal medium under exposure to PBAT. The remaining proteins were present in at least 50% of the conditions, as in the case of the proteins found in only one of the two strains. For instance, 6 out of the 7 polyesterases detected exclusively in the mutant and 2 out of the 3 detected in the wild type, were only found in complete medium. Neither of these proteins exhibited regulation. Results of 3-D structure prediction using modelling by Phyre2 for these proteins are displayed in Table 3, where highest sequence identity and coverage values are observed in matches with acetylcholinesterases.

## Discussion

In this study, for the first time, a secretome screening of the black fungus *Knufia chersonesos* and its non-melanised mutant exposed to PBAT was carried out for the detection of polyesterases with a role in PBAT breakdown. Qualitative and quantitative proteomics along with protein functional analyses and classic methods for the assessment of polymer degradation, allowed to obtaining a deeper insight into the mechanisms used by the fungus to cope with the polymer and ultimately degrade it.

Both the wild type and the mutant were able to hydrolyse PBAT with slightly higher amounts of released molecules detected when cultivated in minimal medium (Fig. 1B). The monomer Ta represented the most abundant compound detected at all experimental conditions and was proven not to be metabolised by the organism. This suggests the involvement of the fungus extracellular enzymes in the cleavage of aromatic ester bonds present in PBAT chains^17,45^, which are reportedly less prone to hydrolysis than the ester bond with aliphatic monomer (adipic acid) as also described for *P. pseudoalcaligenes* polyesterase PpEst^15^. Previous studies on single enzyme hydrolysis process of milled PBAT reported that BTaB and BTa were more abundant than the monomer, as well as that an increase of Ta concentration is given by higher pH than that selected in the current study^7^. Furthermore, the concentrations of released molecules detected in *Knufia* supernatants were quite comparable with those of earlier studies involving purified enzymes^14^ or with other polyesterase screenings, although in the current study the whole secretome was applied^7,18^^,78^. It is therefore suggested that a synergistic action of a number of secreted enzymes with diverse substrate specificities could lie behind the degradation pattern exhibited by the fungus.

A strain-related pattern was observed concerning protein concentration, as the lack of melanin, which affects hyphal pigmentation, also resulted in a reduced complexity of the secretome composition with 1791 and 1070 proteins identified in the Wt and Mut, respectively. From PBAT-exposed and unexposed supernatants a total of 37 polyesterases were identified through protein homology search, the majority of which were predicted to be secreted and were observed in complete medium. 9 enzymes were found to be common to both strains at all cultivation conditions and thus to be expressed regardless of PBAT’s presence and of the nutrient availability in the culture medium (Table 1). Such constitutive production of polyester degrading enzymes reflects *K. chersonesos* observed ability to hydrolyse PBAT at all the established cultivation conditions (Fig. 1B). Quantitative proteomics additionally revealed PBAT-dependent protein regulation at minimal medium condition involving the up-regulation of 7 polyesterases, which is in line with the highest PBAT degradation levels detected in minimal medium. Interestingly, regulation of such classes of proteins was not detected in complete medium. Out of the 7 proteins, 5 showed an up to 3-fold up-regulation in the PBAT-exposed secretome of *K. chersonesos* Wt, while the remaining 2 were detected in the mutant secretome exclusively in presence of PBAT. Protein functional analysis and structure prediction proved the high structural and functional similarity of a number of these proteins with enzymes of already known biotechnological application. The secretory lipase g1109.t1/A0A2K3Q6V2_9HYPO, constitutively expressed by both strains and further increased upon PBAT exposure in the wild type (Fig. 3A), matched the CalA lipase A of *C. antarctica*, reported to hydrolyse long-chained unsaturated fatty acids and to maintain activity at a broad range of temperature and pH^46^. Among the PBAT-up-regulated proteins, also the extracellular cutinase g4295.t1/W2RQJ3_9EURO and the CEH g7566.t1/A0A1Y2EA24_9PEZI have been deemed of special interest based on their similarity with proteins of reported biotechnological use (Fig. 3A; Table 3). Protein g4295.t1, whose expression is exclusively triggered by PBAT in the Mut secretome, exhibited structural similarity to a lipase-like secreted cutinase from *Trichoderma reesei* having specificity for long-chained fatty acids and optimal activity at acidic pH^43^ (Supplementary Table S2; Table 3). g7566.t, regulated in the Wt secretome, showed high alignment coverage to the plant biomass degrading enzyme AoFaeB from *A. oryzae*^36^ as well as to the *I. sakaiensis* MHETase, reported to be involved in a 2-step degradation of highly crystalline PET where it hydrolyses MHET into terephthalate and ethylene glycol. Based on the high similarity of the overall enzymatic structures of the three proteins (Fig. 4), especially encompassing the α/β-hydrolase domain, the catalytic triad and oxyanion hole, it is reasonable to presume that g7566.t1, AoFaeB and MHETase might share similar degradation skills. This may suggest the role of enzyme 7566.t1 in the hydrolysis of BTa, whose structure is similar to that of MHET (only the chain length of the alcohol (ethyl- versus butyl-) is different). Slightly different substrate specificities might be explained by differences at the lid domain level, as shown between AoFaeB and MHETase^38^, and are in line with the low overall sequence similarity (<30% sequence identity) observed between the structurally homologous proteins.

Quantitative proteomics additionally helped elucidating the influence of the culture medium in the regulation of polyesterases, in a strain-dependent manner. While in the wild type the upregulation of target enzymes was mostly recorded in minimal medium, the opposite trend was observed in the mutant. As such, it is reasonable to presume that the Wt secretome relies on a basic set of polyester degrading enzymes whose expression levels are sensitive to nutrient availability and are further modulated by exposure to the polymer. Conversely, the composition of the mutant secretome is qualitatively deeply affected by nutrient availability, with lower diversity being linked to minimal medium. This is possibly the reason behind the detection of PBAT-induced polyester hydrolytic enzymes solely in the mutant strain.

The analysis of significantly enriched GO terms for biological processes elucidated further differences in the secretome rearrangements occurring in Wt and Mut in response to PBAT. Protein functional analysis revealed a secretome enriched in carbohydrate-active enzymes in the wild type. By contrast, the exposure to PBAT drove the mutant into an energy-saving state, however without this affecting the secretome hydrolytic ability (Supplementary Information Section 1.4; Supplementary Figure S5). Regardless of their reaction to PBAT at the secretome level, significant differences in the amount of hydrolysis products released were not observed between the wild type and the mutant during cultivation (21 °C), thereby cell wall melanisation does not seem to play a role in the strain predisposition to polyester degradation. On the contrary, the cultivation conditions did exert a strong effect on the degradation pattern and secretome composition. The lower nutrient availability might therefore have resulted in the fungus attempt to resort to PBAT as an alternative carbon source. Furthermore, the temperature of incubation had a critical role in enhancing the hydrolytic activity of the induced supernatants. Higher levels of released molecules were recorded at both 50 °C and 60 °C, as compared to 21 °C, with uppermost values always linked to low nutrient condition of growth. Between the two tested temperatures, 50 °C triggered the release of the highest amount of monomer and dimer especially in the mutant strain, over a 72 hours long exposure to PBAT (i.e. 610 μM Ta and 45 μM BTa). This might be due to the influence of temperature on the catalytic activity and stability of the enzymes as well as to the increased accessibility of the polymer chains^14^.

These results highlight interesting properties of *K. chersonesos* secretome and suggest their biotechnological application in the field of polymer processing, recycling and degradation. They additionally endorse the use of proteomics as an efficient tool for the detection of novel polyesterases, also in emerging model organisms where an annotated genome is not available. To further our understanding about these enzymes and their PBAT degradation activity, future work shall aim at their characterization in the frame of cloning and expression studies.

## Methods

### **Chemicals and materials**

The commercially available variant of milled PBAT^47^ was investigated for degradation by *K. chersonesos* MA5789 wild type (Wt) and mutant MA5790 (Mut). PBAT had the following properties: thickness of 50 µm, glass transition temperature (Tg) of ─34 °C, melting temperature of 125.3 °C, molecular weight of 65,000 g/mol, Mw/Mn of 3.4 and crystallinity of ~10%^48^. Prior to use, PBAT powder was washed 3x with sterile-filtered 5 g/L Triton X-100 solution, followed by 100 mM Na_2_CO_3_ and subsequently dH_2_O. Each washing step was performed at room temperature under constant stirring for 15 min. Milled PBAT was then left to dry.

The two *K. chersonesos* strains (syn. *K. petricola*, *Sarcynomyces petricola*; order *Chaetothyriales*, family *Trichomeriaceae*^49^) were obtained from the ACBR fungal culture collection of the University of Natural resources and Life Sciences, Vienna, Austria. The wild type had previously been isolated from red sandstone in Ny London, Svalbard, Norway^33^. The pink mutant – whose pigmentation is due to unmasking of carotenoids resulting from melanin synthesis deficiency^50,51^ – originated spontaneously under laboratory conditions. Genome-wide preliminary studies demonstrated that the mutant is isogenic to the wild type. Despite the lack of melanin, yet considered as the main stress protectant in fungi, the mutant exhibits unaltered ability to cope with stress (e.g. desiccation, oligotrophy, thermotolerance)^29^.

### **Exposure of*Knufia chersonesos*Wt and Mut cultures to PBAT**

*K. chersonesos* was cultured in suspension in 2% malt extract (ME, referred to as complete medium, pH 5, 2% malt extract, 2% glucose and 1% peptone) and in 0.2% ME without glucose (referred to as minimal medium, pH 5.5, 0.2% malt extract and 0.1% peptone). Complete and minimal medium were chosen to assess the ability of the fungus to resort to PBAT when supplied as additional or sole carbon source and to detect related changes in polymer degradation. Unexposed cultures were prepared by inoculating 8 mL medium with 50 mg fungal biomass. In exposed cultures, the medium was supplemented with 80 mg milled PBAT (i.e. 1% of final volume). Negative controls were additionally set-up with only medium and polymer to check for microorganisms-independent polymer degradation. All tests were conducted in 6-well-plates with lid (CytoOne, StarLab) producing three biological replicates for each experimental condition (i.e. 3 wells with 8 mL medium/each). An additional identical set of experiments was established for the proteomics analyses. The cultures were kept at 21 °C – the strain’s optimal temperature – with shaking at 100 rpm for 14 days (MaxQ6000, Thermo Fisher Scientific), in order to ensure a sufficiently long incubation with the polymer as well as appropriate amounts of material for further analyses. Following incubation, the cultures supernatants were collected and centrifuged at 4 °C and 4.816 x g for 10 min (Heraeus Megafuge, Thermo Fisher Scientific) to pellet remaining biomass and polymer and thereafter processed for HPLC-based analysis of PBAT released molecules and shotgun proteomics.

### **Analysis of molecules released from PBAT during exposure of fungal cultures**

Following incubation with PBAT, the supernatants were diluted with ice-cold methanol (1:1 v/v) in order to precipitate the enzymes and acidified to pH 3.0 with 1 M HCl. The samples were centrifuged at 18.213 x g (5427 R, Eppendorf) for 15 min at 4 °C, then filtered through 0.45 μm nylon syringe filters and thereafter analysed by a HPLC-DAD system consisting of a 1290 Infinity II LC (Agilent Technologies), coupled with a reversed phase column C18 (Poroshell 120 EC-C18 2,7 µm 3.0 × 150 mm), at a flow rate of 0.4 mL/min^15,19^. The PBAT hydrolysis products were separated using a nonlinear gradient according to Quartinello *et al*.^2^ with some modifications (i.e. solvent A: H_2_O, solvent B: methanol, solvent C: formic acid; solvent C was kept at 10% constantly; 0–13 min 15% B; 13–30 min 15–40% B; 30–35 min 40–90% B; 35–46 min 90–15% B; 46–60 min 15% B) and detected with a photodiode array detector (Agilent Technologies) at the wavelength of 245 nm. The expected released molecules (Fig. 1a) bis(4-hydroxybutyl) terephtalate (BTaB), mono(4- hydroxybutyl) terephthalate (BTa) and terephthalic acid (Ta) were quantified using external calibration curves in the range 0.001–0.5 mM. Values from controls without mycelium added were subtracted from the results. The calculated concentrations were plotted on graph by using Origin Pro software v.9.5 (https://www.originlab.com/origin).

### **Hydrolytic activity of PBAT-exposed supernatants at higher temperatures**

Two millilitres of PBAT-exposed sterile filtered cell-free supernatant from *K. chersonesos* Wt and Mut and from both complete and minimal medium were further incubated at 50 and 60 °C with 10 mg of milled PBAT. Samples were collected at different time points – i.e. 0, 24, 48 and 72 hours – and thereafter precipitated with methanol and quantified by HPLC. Each experiment was performed using 3 biological replicates per condition.

### **Metabolism of terephthalic acid**

In order to verify *K. chersonesos* ability to metabolise Ta, both strains were cultured in suspension in complete and minimal medium supplemented with 1% Ta in final volume (w/V). Negative controls with only medium and the monomer were additionally set-up in order to check for microorganisms-independent changes in Ta concentration. Samples were collected after 0, 3, 6 and 14 days and processed and analysed by HPLC as described earlier. The Ta metabolism was assessed by monitoring changes in its concentration over the incubation time using the value at timepoint 0 as reference. For each timepoint 3 biological replicates were used.

### **Sample preparation for proteomic analysis**

Eight-millilitres of the supernatant were mixed with protease inhibitors (40 μL:1 mL v/v, Complete™, Sigma-Aldrich) and spun at 7500 × g (Heraeus Megafuge, Thermo Fisher Scientific) and at 4 °C for 15 min in order to remove traces of cellular debris. Each sample was then transferred to a Vivaspin PES 15 R 5 kDa cut-off spin filter device (Sartorius) and centrifuged at 6000 × g and at 4 °C to achieve a 20x sample concentration. Proteins were precipitated overnight by adding five volumes ice-cold 0.1 M ammonium acetate in methanol to the concentrated supernatants. Protein pellets were obtained by centrifugation (7.500 × g, 4 °C, 30 min), washed and re-solubilised according to Tesei *et al*.^26^. Protein concentration was determined by the Qubit protein assay (concentration range 0.0125–5 μg/μl; Thermo Fisher Scientific) after dilution of the samples to reduce interference by melanin, solvents and detergents. For the rapid fingerprinting and assessment of sample quality equal amounts of the protein extracts were separated on 10 wells NuPAGE 12% Tris Glycine gels (Invitrogen). Samples were run at 125 V and 30 mA and protein bands were visualized by silver staining after gel fixation in 40% ethanol and 10% acetic acid.

The protein digestion was performed following a standard enhanced, filter-aided sample preparation protocol (FASP)^52^ with some modifications^53^. Briefly, twenty-micrograms protein were reduced using 20 mM dithiothreitol (37 °C, 30 min) and alkylated with 500 mM iodoacetamide (25 °C, 30 min), then filled up to 200 µl with 8 M urea in 50 mM Tris buffer and transferred into a Centrifugal Device (10 kDa cut-off, Merck Millipore). On-filter digestion was performed overnight at 37 °C with 0.1 μg/μl Trypsin/LysC protease mix (Promega). The peptides were eluted from the filter with 3 changes of 50 μl 50 mM Tris, each (14 000 × g, 20 min). Sample desalting was achieved with a Pierce C18 Spin Column (Thermo Fisher Scientific) according to manufacturer’s instructions. The purified peptides were dissolved in 0.1% trifluoroacetic acid (TFA) prior to mass spectrometry analysis.

### **HPLC-MS/MS analysis**

A total of 600 ng of the samples were injected on a nano-HPLC Ultimate 3000 system (Dionex, Thermo Fisher Scientific) equipped with a 25 cm C18 Acclaim Pepmap column (Dionex, 75 µm inner diameter, 2 µm particle size, 100 Å pore size). The sample pre-concentration and desalting were accomplished on an Acclaim PepMap, 5 µm, 300 µm × 5 mm μ-precolumn (Thermo Fisher Scientific) using 2% acetonitrile (ACN) in HPLC water with 0.05% TFA, with a flow rate of 5 µl/min. Peptide separation was carried out starting with 96% mobile phase A (0.1% formic acid in HPLC water) and 4% of mobile phase B (80% ACN in HPLC water with 0.1% formic acid), then increasing B to 31% in 30 minutes and to 44% in additional 5 minutes. The gradient was followed by a washing step with 95% solvent B. Flow rate was 300 nL/min.

The separated peptides were directly analysed in a high-resolution Q Exactive HF Orbitrap mass spectrometer (Thermo Fisher Scientific) for both identification and label-free quantification, according to Shikov *et al*.^53^. Mass spectrometry full scans were performed in the ultrahigh-field Orbitrap mass analyser in the ranges 350–2000 m/z with a resolution of 60000, the maximum injection time (MIT) was 50 ms and the automatic gain control (AGC) was set to 3e^6^. The top 10 intense ions were subjected to Orbitrap for further fragmentation via high-energy collision dissociation (HCD) activation over a mass range between 200 and 2000 m/z at a resolution of 15000 with the intensity threshold at 4e^4^. The ions with charge state +1, +7, +8 and larger than +8 were excluded. Normalised collision energy (NCE) was set at 28. For each scan, the AGC was set at 5e^4^ and the MIT was 50 ms. Dynamic exclusion of precursor ion masses over a time window of 50 s was used to suppress repeated peak fragmentation. Two technical replicates (LC-MS/MS run) were performed for each of the three biological replicates (48 runs in total) in order to counteract additional variations introduced by the methodology and to increase the number of the identified proteins.

### **Database search**

Because of the unavailability of a full genome annotation for *K. chersonesos*, the MS/MS spectra were analysed by searching a database of predicted proteins obtained by the *ab initio* translation of the fungus genome using Augustus^54,55^. Due to phylogenetic proximity, *Exophiala dermatitidis* CBS525.76 annotated genome (GCA_000230625.1, NCBI, 239 contigs, 26,376,767 bp) was used as training database for homology-based identification. The genome sequence of *K. chersonesos* MA5789 Wt (GCA_002319055.1, assembly ASM231905v1, NCBI)^33^ consisting of 388 contigs, 27,759,230 bp was thus translated into 9847 proteins. To reject all identified proteins stemming from other sources than the secretome of *K. chersonesos*, the so-called cRAP database (https://www.thegpm.org/crap/) and the UniProt database of barley were used. Common contaminants and malt proteins were excluded for the statistical analysis. Searches were performed with Sequest search engine (Proteome Discoverer Software 2.3.0.523, Thermo Fisher Scientific) with the following parameters: trypsin as enzyme, two max. missed cleavage sites, MS tolerance of 20 ppm, MS/MS mass tolerance of 0.02 Da, carbamidomethylation of cysteine as fixed modification, oxidation of methionine and acetylation of N-terminus as variable modifications; decoy database search with FDR set to 1% (strict) on PSMs level; Percolator node for PSMs filtering and validation of the identifications. Only proteins with at least two peptides per protein were considered. Proteomics data and the database of predicted proteins have been deposited to the ProteomeXchange Consortium (http://proteomecentral.proteomexchange.org) via the PRIDE partner repository with the dataset identifier PXD014026^56^.

### **Protein identification, quantification and bioinformatics analysis**

Protein identifications and functional insights were obtained searching for sequence homologs using HMMER 3.2. (http://hmmer.org)^57^ together with PFAM (https://www.ebi.ac.uk/Tools/hmmer/search/phmmer; Gathering threshold)^58^. Proteins having domains structural or sequence similarity were assigned to the same PFAM clan, for which an e-value threshold was defined^59^. To characterize proteins in respect of the biological process they are involved in, Gene Ontology (GO) terms were assigned to domains using Blast2GO PRO (https://www.blast2go.com/)^60^. The sequences were blasted (BLASTP, NCBI BLAST, E-Value 1.0E-3) and the blast hits were mapped and annotated with GO Terms using the GO database (http://geneontology.org, E-Value 1.0E-6, Filter GO by Taxonomy: taxa: 4751, Fungi). The annotations were validated based on the True-Path-Rule by removing all redundant terms to a given sequence. A GO-Slim analysis was run to summarize the GO annotation using the *Aspergillus* slim. Existing GO terms were additionally mapped to enzymes codes, when possible.

To confirm the identity of all proteins detected as hydrolases, a search for homology was further performed in the UniProtKB database (http://www.uniprot.org/blast; BLASTP parameters: E-Threshold: 10; matrix BLOSUM62). In case of blast results where the most significant match was represented by an uncharacterized protein, the first match in the list of homologous proteins where a protein ID was available was considered. HMMER and BLASTP results where compared to check for consistency in the protein IDs, especially when the identity values associated to the homologous protein where lower than 50%. The list of all identified hydrolases was thereafter used as database in the quantitative analysis, along with the above-mentioned protein databases.

For the quantification analysis the following parameters were applied: only unique peptides were used, and the precursor abundance was based on the intensity. Normalization was carried out on total peptide amount, scaling on all average. The protein abundances were calculated using summed abundances. The ratio calculation for the t-test was done pairwise excluding modified peptides. Experimental sets were created to compare all the established cultivation conditions (Table 2). No imputation was performed. All proteins deemed as regulated had an abundance adjusted *p*-value lower than 0.05 and a fold change of at least 2. Principal component analysis (PCA) and hierarchical clustering (HC) were further performed using the normalized abundance values to check the clustering of different biological replicates as well as to search for groups of co-varying proteins.

REVIGO^61^ was used to summarize the list of overrepresented biological process GO terms associated to the groups of proteins with changed abundance, by clustering semantically close terms (categorization threshold: 0.5, semantic similarity measure: Sim Real). Information about the protein subcellular localization was obtained using BUSCA (http://busca.biocomp.unibo.it)^62^. The computation of theoretical pI (isoelectric point) and Mw (molecular weight) was performed using the ExPASy Compute pI/Mw tool (https://web.expasy.org/compute_pi/)^63^. Further characterization of the enzymes of interest was carried out using secondary (coupled to fold-recognition) and three-dimensional structure prediction. 3-D models were generated using, Phyre V 2.0^2,64^ in intensive mode, by applying the crystal structure of homologous proteins as templates. Graphic representations of the 3-D structures and of proteins catalytic sites were elaborated with PyMOL Molecular Graphics System (v. 1.3, Schrödinger, LLC).

## Supplementary information

Supplementary information10.

## Data Availability

The proteomics datasets generated during the current study are available in the ProteomeXchange Consortium (http://proteomecentral.proteomexchange.org) via the PRIDE partner repository with the dataset identifier PXD014026.
