# Author Correction: Shotgun proteomics reveals putative polyesterases in the secretome of the rock-inhabiting fungus *Knufia chersonesos*

**DOI:** 10.1038/s41598-020-70778-5

**Published:** 2020-10-02

**Authors:** Donatella Tesei, Felice Quartinello, Georg M. Guebitz, Doris Ribitsch, Katharina Nöbauer, Ebrahim Razzazi-Fazeli, Katja Sterflinger

**Affiliations:** 1 Institute of Microbiology and Microbial Biotechnology, University of Natural Resources and Life Sciences, Muthgasse 18, 1190 Vienna, Austria; 2 Institute of Environmental Biotechnology, University of Natural Resources and Life Sciences, Konrad Lorenz Strasse 20, 3430 Tulln, Austria; 3 Austrian Centre of Industrial Biotechnology, Konrad Lorenz Strasse 20, 3430 Tulln, Austria; 4 VetCore Facility for Research, University of Veterinary Medicine, Veterinärplatz 1, 1210 Vienna, Austria

Correction to: *Scientific Reports* 10.1038/s41598-020-66256-7, published online 17 June 2020

The original version of this Article contained errors in the Abstract.

“Protein functional analysis and structure prediction indicated similarity of these enzymes to microbial polyesterases of known biotechnological use such as MHETase from *Ideonella sakaiensis* and CalA from *Candida albicans*.”

now reads:

“Protein functional analysis and structure prediction indicated similarity of these enzymes to microbial polyesterases of known biotechnological use such as MHETase from *Ideonella sakaiensis* and CalA from *Candida antarctica*.”

Additionally, there were typographical errors in the Article. In the Results section, under the subheading ‘Differential abundance and characterization of PBAT-regulated polyesterases’,

“The 2 enzymes found in the mutant instead, were solely detected in the PBAT-exposed secretome (i.e. 44295t1 and g8915.t1).”

now reads:

“The 2 enzymes found in the mutant instead, were solely detected in the PBAT-exposed secretome (i.e. 4295.t1 and g8915.t1).”

Under the subheading ‘Modelling of the carboxylic ester hydrolase g7566.t1’,

“This is consistent with structural observations on MHETase, which described the lid domain as the major difference to the closely related tannase and feruloyl esterases^38^.”

now reads:

“This is consistent with structural observations on MHETase, which described the lid domain as the major difference to the closely related tannases and feruloyl esterases^38^.”

In the Discussion section,

“g7566.t, regulated in the Wt secretome, showed high alignment coverage to the plant biomass degrading enzyme AoFaeB from *A. oryzae*^36^ as well as to the *I. sakaiensis* MHETase, reported to be involved in a 2-step degradation of highly crystalline PET where it hydrolyses MHET into terephthalate and ethylene glycol.”

now reads:

“g7566.t1, regulated in the Wt secretome, showed high alignment coverage to the plant biomass degrading enzyme AoFaeB from *A. oryzae*^36^ as well as to the *I. sakaiensis* MHETase, reported to be involved in a 2-step degradation of highly crystalline PET where it hydrolyses MHET into terephthalate and ethylene glycol.”

These errors have now been corrected in the PDF and HTML versions of the Article.

