# Supplementary material for: Shotgun proteomics reveals putative polyesterases in the secretome of the rock-inhabiting fungus Knufia chersonesos
Source: Sci Rep. 2020 Jun 17;10:9770. doi: 10.1038/s41598-020-66256-7 (PMC7299934; doi:10.1038/s41598-020-66256-7)
Supplement: Supplementary file 1 — Supplementary information10. [file 41598_2020_66256_MOESM1_ESM.docx]

Supplementary information to:

Shotgun proteomics reveals putative polyesterases in the secretome of the rock-inhabiting fungus *Knufia chersonesos*.

Donatella Tesei ^a*^, Felice Quartinello ^b^, Georg M. Guebitz ^b,c^, Doris Ribitsch ^b,c^, Katharina Nöbauer ^d^, Ebrahim Razzazi-Fazeli ^d^, Katja Sterflinger^a^.

^a^ Institute of microbiology and microbial biotechnology, University of Natural Resources and Life Sciences, Muthgasse 18 A, 1190 Vienna, Austria

^b^ Institute of Environmental Biotechnology, University of Natural Resources and Life Sciences, Konrad Lorenz Strasse 20, 3430 Tulln an der Donau, Austria

^c^ Austrian Centre of Industrial Biotechnology, Konrad Lorenz Strasse 20, 3430 Tulln, Austria

^d^ VetCore Facility for Research, University of Veterinary Medicine, Vienna, Veterinärplatz 1, 1210 Vienna, Austria

**^*^ Corresponding author:** Donatella Tesei, Email: [donatella.tesei@boku.ac.at](mailto:donatella.tesei@boku.ac.at)

Number of pages: 35

Number of figures: 5

Number of Tables: 4

**Table of Contents**

**1 Supplementary Results3**

1.1 Characterization of the culture supernatant

Secretome profiling of *K. chersonesos* Wt and Mut, Fig. S13

Experimental overview of the hydrolysis of PBAT by *K. chersonesos* extracellular enzymes, Fig.S24

1.2 Proteome screening of unexposed and PBAT-exposed supernatants

Number of identified secreted proteins and of hydrolases in *K. chersonesos* Wt and Mut, Table S15

Homologues of *K. chersonesos* polyesterases, detected in the UniProtKB database, Table S26

1.3 Overview of secretome quantitative analysis

PCA Analysis showing the clustering of the secretomes, Fig. S38

Heatmaps displaying the abundance pattern of the identified proteins, Fig. S49

1.4 Protein differential abundance10

*K. chersonesos* Wt, control and PBAT- supplemented cultivation in minimal medium10

*K. chersonesos* Mut, control and PBAT-supplemented cultivation in minimal medium11

*K. chersonesos* Wt and Mut exhibit opposite responses to PBAT at the secretome level12

Predicted sub-cellular localizations of the modulated secreted proteins, Table S313

Biological processes GO terms associated to the modulated secreted proteins, Fig. S529

Relative abundance of proteins involved in carbohydrate and lipid metabolism and in response to stress, Table S430

1.5 Sequences of the polyesterases of interest (as listed in Table 3) 33

**Supplementary Figure S1**

**
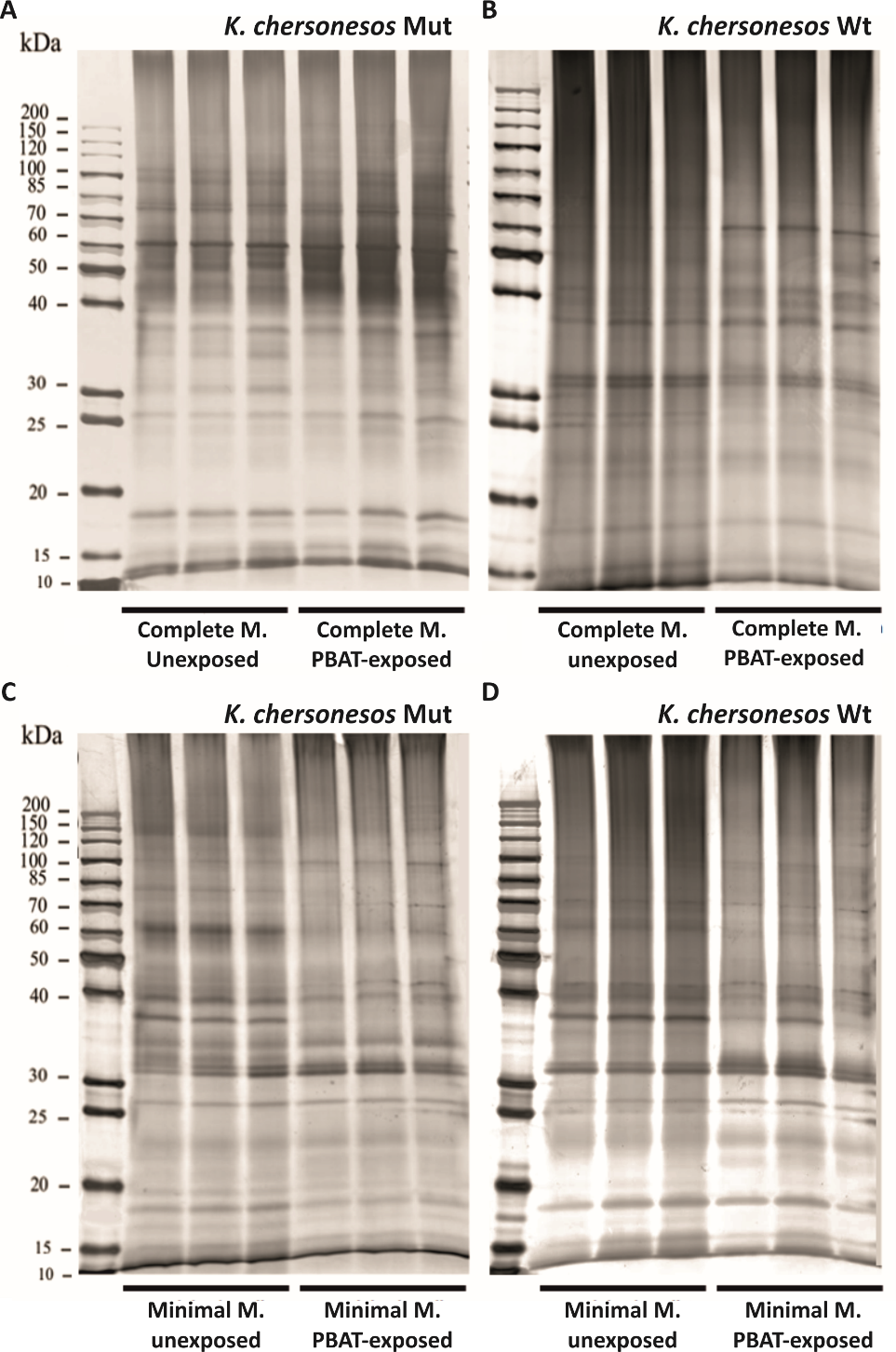
**

**Supplementary Figure S1_** Secretome profiling of *K. chersonesos* Mut and Wt without PBAT and under PBAT exposure. Protein extracts were obtained as described in the Methods session and separated by SDS-PAGE on a 12% Tris-Glycine gel, after precipitation. Protein amount: 2 µg, Ladder: 200-10 kDa. Bands were visualized using silver staining. (a, c) *K. chersonesos* MA5790, Mut; (b, d) *K. chersonesos* MA5789, Wt. The dark background visible in *K. chersonesos* Wt samples is due to the interference of melanin – released into the culture supernatant – with the silver nitrate. Minimal M.: minimal medium; Complete M.: complete medium. Gel images were acquired using Typhoon FLA 9500 (GE Healthcare) and the figure was created with the Corel DRAW Graphics Suite 2019 software (<https://www.coreldraw.com/de/>).

**Supplementary Figure S2**

**
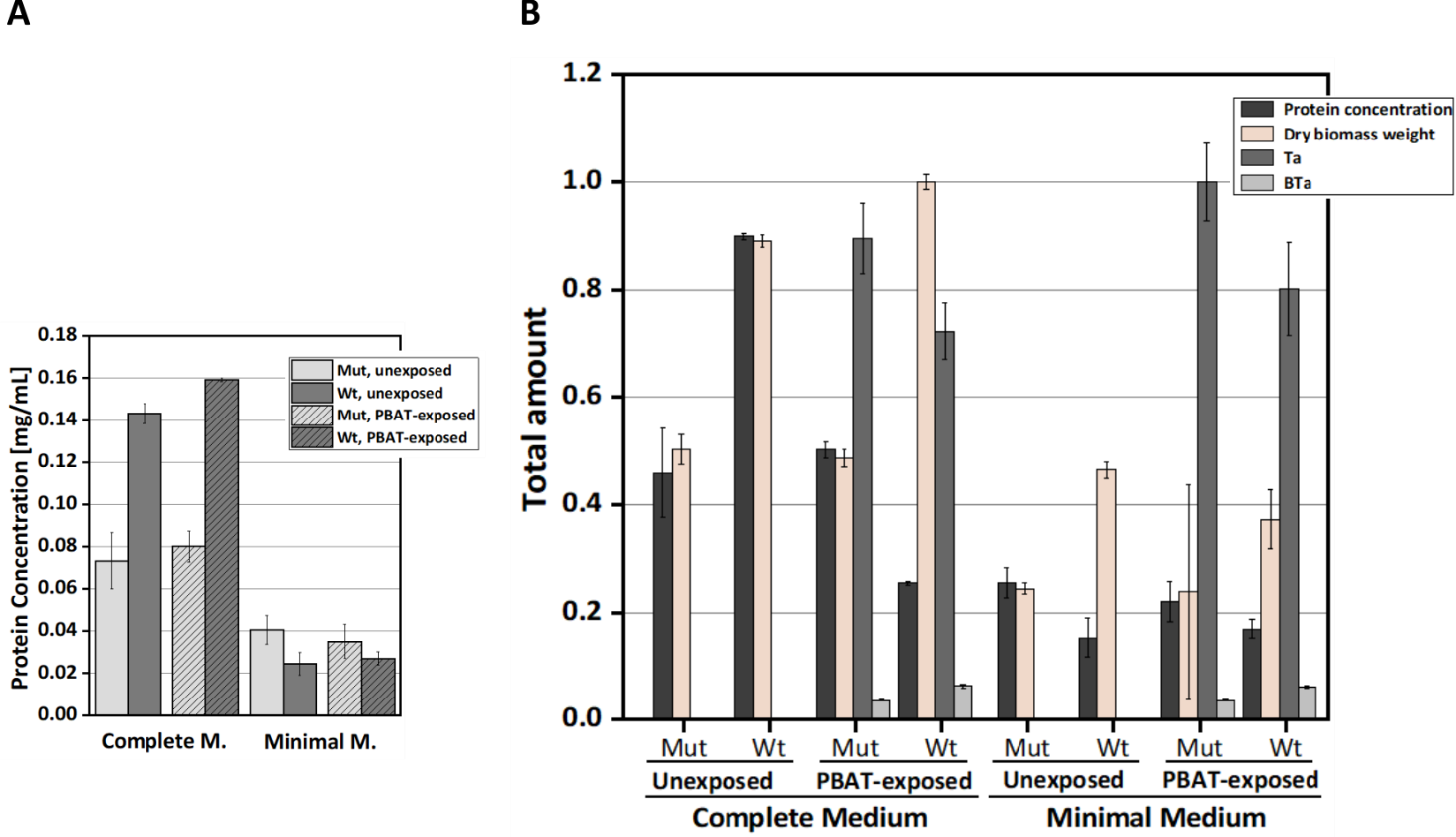
**

**Supplementary Figure S2_** (a) Concentrations of the proteins secreted in the culture supernatants of *K. chersonesos* Wt MA5789 and Mut MA5790 as determined by the Qubit protein assay. (b) Experimental overview of the hydrolysis of PBAT by *K. chersonesos* extracellular enzymes (protein concentrations, biomass weight and concentrations of the released products Ta and BTa. The datapoints were normalized using min-max normalization. Each bar represents the average of three replicates ± standard deviation (error bars). All graphs were created using Origin Pro v 9.5 (<https://www.originlab.com/origin>).

**Supplementary Table S1**

**Supplementary Table S1_** Number of proteins identified in the culture supernatant at each experimental condition. The total number of proteins as well as the number of protein subgroups are displayed.

| **Sample Name** | **Total No. of identified proteins ^a^** | **No. of malt and cRAP ^b^ proteins** | **No. of identified proteins** | **No. of hydrolases** |
| --- | --- | --- | --- | --- |
| Wt^c^, Minimal medium, unexposed | 1437 | 27 | 1410 | 222 |
| Wt, Minimal medium, PBAT-exposed | 1106 | 22 | 1084 | 193 |
| Mut^d^, Minimal medium, unexposed | 702 | 12 | 690 | 141 |
| Mut, Minimal medium, PBAT-exposed | 450 | 9 | 441 | 107 |
| Wt, Complete medium, unexposed | 1460 | 27 | 1433 | 221 |
| Wt, Complete medium, PBAT-exposed | 1441 | 23 | 1418 | 219 |
| Mut, Complete medium, unexposed | 831 | 17 | 814 | 177 |
| Mut , Complete medium, PBAT-exposed | 814 | 19 | 795 | 169 |

^a^ Total number of proteins including common protein contaminants (i.e. cRAP) and malt proteins (i.e. barley database, UniProt)

^b^ cRAP: Repository of Adventitious Proteins (database of proteins detected in proteomics experiments and present either by accident or through unavoidable contamination of protein samples).

^C^ *Knufia chersonesos,* wild type strain

^d^ *Knufia chersonesos,* mutant strain

**Supplementary Table S2**

**Supplementary Table S2_** Homologues of *K. chersonesos* proteins identified as esterases, cutinases and lipases, detected in the UniProtKB database through homology search (BLASTP algorithm). Only the matching proteins with known identity and the highest max score are displayed. The predicted protein subcellular localisations are also shown.

| ***Protein* accession No. ^a^** | **UniProtKb**  **accession**  **No.** | **UniProtKb Protein name** | **Max**  **Score** | **Identity**  **(%)** | **Expected**  **value** | **Predicted Localisation** | **BUSCA Score** | | |
| --- | --- | --- | --- | --- | --- | --- | --- | --- | --- |
| g1109.t1 | A0A2K3Q6V2_9HYPO | Secretory lipase (*Tolypocladium capitatum*) | 1,134 | 48.8 | 3E-148 | Extracellular space | | 1 |  |
| g1329.t1 | A0A0D2C9P1_9EURO | Carboxylic ester hydrolase (*Exophiala xenobiotica*) | 1,380 | 57 | 0 | Extracellular space | | 1 |  |
| g1587.t1 | H6BMJ0_EXODN | Triacylglycerol lipase (*Exophiala dermatitidis*, CBS 525.76) | 1,368 | 69.1 | 0 | Extracellular space | | 0.99 |  |
| g2066.t1 | A0A0N1HTM9_9EURO | Esterase LovG (*Phialophora attae*) | 705 | 59.7 | 4.5E-90 | Nucleus | | 1 |  |
| g2279.t1 | A0A1J9RJA8_9PEZI | Carboxylic ester hydrolase (*Diplodia corticola*) | 1,819 | 63.0 | 0 | Extracellular space | | 0.99 |  |
| g2917.t1 | M3AEN1_PSEFD | Carbohydrate esterase family 1 protein (*Pseudocercospora fijiensis, CIRAD86)* | 873 | 52.1 | 2.6E-113 | Extracellular space | | 0.99 |  |
| g2930.t1 | W9VX79_9EURO | Carboxymethylenebutenolidase (*Cladophialophora yegresii CBS 114405)* | 1,182 | 80.1 | 4.9E-162 | Cytoplasm | | 0.81 |  |
| g3032.t1 | A0A0D2FB23_9EURO | Phospholipase (*Phialophora americana*) | 3,447 | 53.2 | 0 | Nucleus | | 1 |  |
| g3128.t1 | W9WP45_9EURO | Protein ssh4 (*Cladophialophora psammophila CBS 110553)* | 1,848 | 75.9 | 0 | Endomembrane system | | 0.73 |  |
| g3531.t1 | H6BST6_EXODN | S-formylglutathione hydrolas (*Exophiala dermatitidis* CBS 525.76) | 1,320 | 83.3 | 0 | Nucleus | | 1 |  |
| g3802.t1 | H6BS65_EXODN | Triacylglycerol lipase (*Exophiala dermatitidis* CBS 525.76) | 815 | 53.8 | 1.8E-105 | Extracellular space | | 1 |  |
| g3878.t1 | A0A0F4GY99_9PEZI | Thioesterase family protein (*Zymoseptoria brevis*) | 593 | 66.7 | 6.8E-76 | Nucleus | | 1 |  |
| g4102.t1 | A0A1C1CIG2_9EURO | Isoamyl acetate-hydrolyzing esterase 1 like protein *(Cladophialophora carrionii*) | 534 | 44.6 | 3.1E-64 | Nucleus | | 1 |  |
| g4295.t1 | W2RQJ3_9EURO | Cutinase *(Cyphellophora europaea CBS 101466*) | 506 | 48.4 | 4.2E-61 | Extracellular space | | 1 |  |
| g4612.t1 | A0A1L7WLC8_9HELO | Carboxylic ester hydrolase (*Phialocephala subalpina*) | 2,077 | 75 | 0 | Anchored component of plasma membrane | | 0.75 |  |
| g4621.t1 | A0A3M7ILY7_HORWE | Cutinase *(Hortaea werneckii)* | 536 | 47.6 | 1.1E-59 | Endomembrane system | | 0.75 |  |
| g5276.t1 | H6C0F4_EXODN | Ubiquitin thiolesterase (*Exophiala dermatitidis* CBS 525.76) | 2,211 | 73.1 | 0 | Nucleus | | 1 |  |
| g5383.t1 | A0A1L7X3A1_9HELO | Carboxylic ester hydrolase (*Phialocephala subalpina*) | 1,882 | 70.7 | 0 | Extracellular space | | 0.99 |  |
| g5594.t1 | H6C8P0_EXODN | Triacylglycerol lipase (*Exophiala dermatitidis*, CBS 525.76) | 542 | 39.7 | 3.5E-63 | Nucleus | | 1 |  |
| g5645.t1 | A0A0D2IP65_9EURO | Carboxylic ester hydrolase (*Rhinocladiella mackenziei* CBS 650.93) | 1,625 | 54.7 | 0 | Nucleus | | 1 |  |
| g 5761.t1 | A0A1L7XT81_9HELO | Carboxylic ester hydrolase (*Phialocephala subalpina*) | 1,312 | 51.6 | 5e-174 | Plasma Membrane | | 0.83 |  |
| g5776.t1 | A0A1C1CG26_9EURO | Putative esterase C31F10.02 (*Cladophialophora carrionii*) | 588 | 64.7 | 5.2E-74 | Extracellular space | | 1 |  |
| g6131.t1 | H6C9K5_EXODN | Esterase/lipase *(Exophiala dermatitidis, CBS 525.76)* | 907 | 68.9 | 4.9E-121 | Cytoplasm | | 0.7 |  |
| g6247.t1 | A0A0D2AQ38_9EURO | Lysophospholipase *(Exophiala oligosperma)* | 2,090 | 61 | 0 | Anchored component of plasma membrane | | 1 |  |
| g6560.t1 | H6C4Y8_EXODN | Carboxylesterase (*Exophiala dermatitidis* CBS 525.76) | 420 | 31.1 | 2.2e-45 | Nucleus | | 1 |  |
| g6652.t1 | A0A0K0KDL6_9PEZI | Carboxylic ester hydrolase (*Daldinia eschscholtzii*) | 1,638 | 56.2 | 0 | Extracellular space | | 0.98 |  |
| g723.t1 | A0A194VTH9_9PEZI | Carboxylic ester hydrolase (*Valsa mali*) | 1,917 | 61.5 | 0 | Extracellular space | | 1 |  |
| g7247.t1 | A0A0N1HQ42_9EURO | PI-PLC X domain-containing protein (*Phialophora attae*) | 1,194 | 59.0 | 3.6E-160 | Anchored component of plasma membrane | | 0.55 |  |
| g7566.t1 | A0A1Y2EA24_9PEZI | Carboxylic ester hydrolase (*Pseudomassariella vexata*) | 1,837 | 58.1 | 0 | Extracellular space | | 1 |  |
| g7567.t1 | A0A2V1CGY1_9HELO | Para-nitrobenzyl esterase (Cadophora sp. DSE1049) | 1,945 | 70.3 | 0 | Extracellular space | | 0.99 |  |
| g7569.t1 | A0A0D2AG04_9PEZI | Cutinase (*Verruconis gallopava*) | 704 | 54.0 | 3.3E-90 | Extracellular space | | 1 |  |
| g762.t1 | A0A0G2E0T4_9PEZI | Putative erythromycin esterase (*Diplodia seriata)* | 1,590 | 65.7 | 0 | Nucleus | | 1 |  |
| g7983.t1 | A0A1L9SZB3_9EURO | Carboxylic ester hydrolase (*Aspergillus sydowii* CBS 593.65) | 1,084 | 50.8 | 1.7E-141 | Extracellular space | | 0.99 |  |
| g8915.t1 | A0A1L7WTP0_9HELO | Carboxylic ester hydrolase (*Phialocephala subalpina)* | 1,256 | 44.5 | 1.3E-164 | Anchored component of plasma membrane | | 1 |  |
| g8978.t1 | A0A1Q8RG26_9PEZI | Carboxylic ester hydrolase (*Colletotrichum chlorophyti*) | 2,111 | 69.3 | 0 | Endomembrane system | | 0.61 |  |
| g9204.t1 | A0A072NTL7_9EURO | Coesterase *(Exophiala aquamarina CBS 119918)* | 1,937 | 62.8 | 0 | Extracellular space | | 1 |  |
| g9456.t1 | A0A1C1CYP6_9EURO | Putative esterase C31F10.02 (*Cladophialophora carrionii)* | 574 | 70.5 | 1.9E-73 | Cytoplasm | | 0.7 |  |

**^a^** Protein accession number in the *K. chersonesos* database of ab initio translated proteins

**Supplementary Figure S3**





**Supplementary Figure S3_** PCA Analysis showing the clustering of the secretomes (2 technical replicates for each of the 3 biological replicates) of *K. chersonesos* Wt and Mut. Data source: the identified proteins. (a) Complete medium condition; (b) Minimal medium condition; (c) *K. chersonesos* Wt MA5789; (d) *K. chersonesos* Mut MA5790. Each data point represents a secretome and the subset of associated proteins, whose ratios varied fold change of 2 or higher (p≤0.05). Values of variance for component 1 and 2 are shown. Minimal M.: minimal medium; Complete M.: complete medium. PCA plots were generated using Proteome Discoverer Software 2.3.0.523.

**Supplementary Figure S4**

**
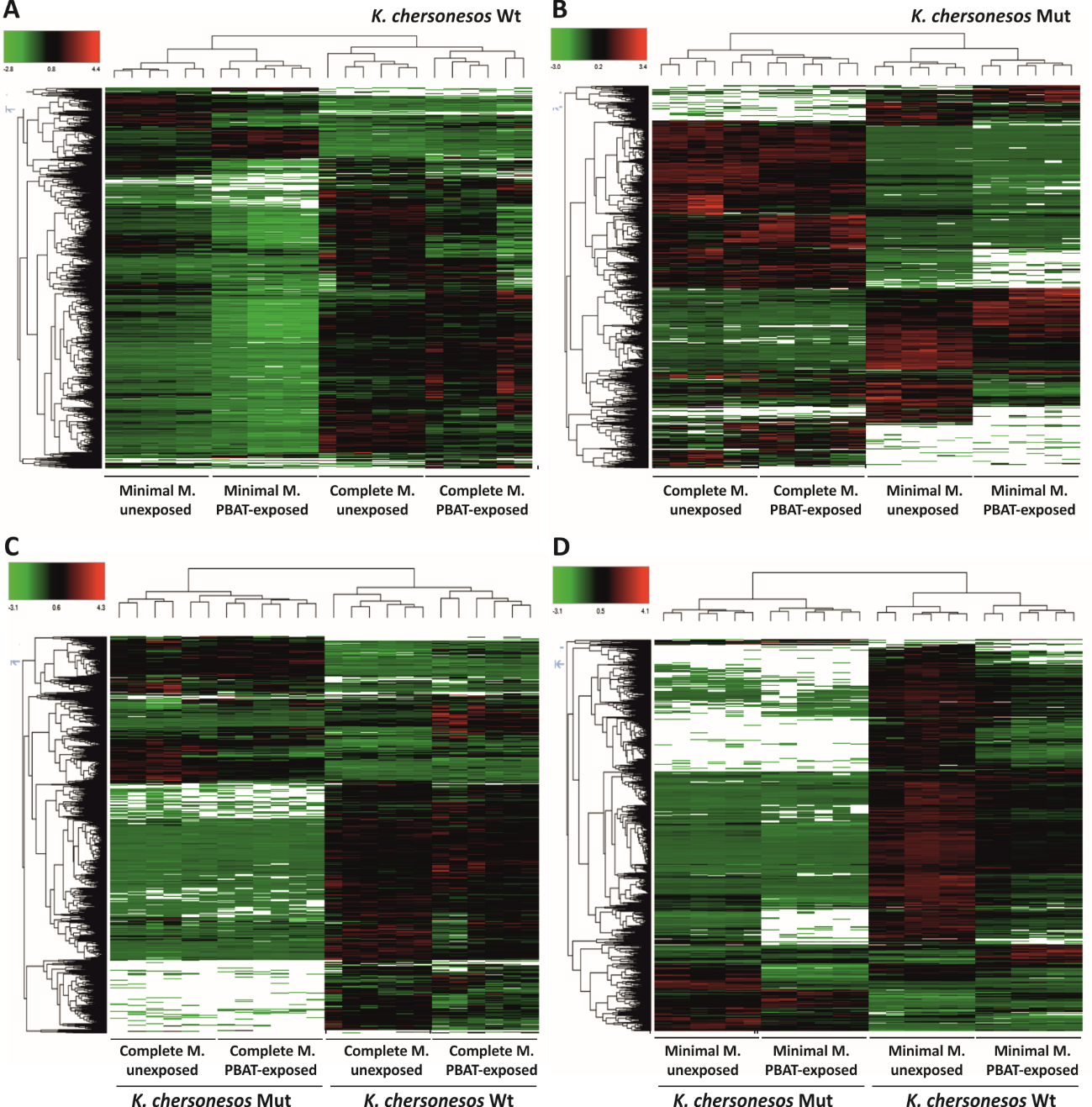
**

**Supplementary Figure S4_** Heatmaps displaying the abundance pattern of the identified proteins. Dendrograms of co-varying proteins are shown for (a) *K. chersonesos* Wt MA5789, (b) *K. chersonesos* Mut MA5790; (c) complete medium condition; (d) minimal medium condition. Normalized abundances are displayed as a heatmap where red indicates increased abundance and green decreased abundance. White boxes indicate missing values. A total of 6 runs – 3 biological replicates and 2 technical replicates each – were performed for each sample. Minimal M.: minimal medium; Complete M.: complete medium. Heatmaps were generated using Proteome Discoverer Software 2.3.0.523.

**Protein differential abundance**

*K. chersonesos*Wt, unexposed and PBAT-exposed cultivation in minimal medium

The quantitative analysis of PBAT-exposed v/s unexposed secretome of *K. chersonesos* Wt from minimal medium resulted in the identification of 67 proteins with increased and 55 proteins with decreased abundance in the exposed, relative to the unexposed secretome. Around 70% of the regulated proteins had a predicted extracellular localization (Supplementary Table S3), the remaining 30% was instead deemed as intracellular.

Distribution of overrepresented biological process GO terms among differentially expressed proteins is displayed in Supplement. Fig. S5a. Most of proteins exhibiting increased abundance upon PBAT exposure were involved with lipid and carbohydrate metabolism (35% and 12% of GO terms associated to the upregulated proteins, respectively), response to chemicals (11%), tRNA thio-modification (6.5%) and organelle organization (5.5%). A number of hydrolases associated to lipid and carbohydrate metabolism exhibited differential levels in presence of PBAT (Supplementary Table S4). The extracellular glycoside hydrolase 16 (AA1C1D0J4_9EURO) and xyloglucan-specific endoglucanase (C9SYD2_VERA1) were uniquely detected in PBAT-supplemented cultivations. Glucan 1,3-beta-glucosidase (W9WT35_9EURO), endo-1,3(4)-beta-glucanase (W9XFN3_9EURO), endoglucanase (H6C6J4_EXODN), the extracellular cell wall glucanase Crf1 (H6BNZ6_EXODN) and 1,3-beta-glucanosyltransferases (A0A0D2C082_9EURO, A0A0D2FAU6_9EURO and A0A0D2F228_9EURO) involved in degradation or elongation of β-glucan chains ^54^, showed higher levels in the treatment. The same for mannan endo-1,6-alpha-mannosidase (A0A0D2KI27_9EURO) and the glycerophosphoryl diester phosphodiesterase (A0A0D2CPX4_9EURO), known to play a role in cell wall organization and biogenesis ^55^. Conversely, decreased levels of glycoside hydrolases with alpha-L-arabinofuranosidase activity were observed in the treatment for arabinan endo-1,5-alpha-L-arabinosidase (A0A364MWV0_9PLEO), the non-reducing end alpha-L-arabinofuranosidase BoGH43B (A0A0S7DK79_9EURO) and for an additional endoglucanase (H6C6J4_EXODN), the latter solely detected in the control secretome. Lysophospholipase (A0A0D2AQ38_9EURO), carboxylic ester hydrolase (A0A1Y2EA24_9PEZI), cutinase (A0A0D2AG04_9PEZI), carboxylic ester hydrolase (A0A1J9RJA8_9PEZI) and secretory lipase (A0A2K3Q6V2_9HYPO), lipolytic/esterolytic enzymes reported to being able to catalyse the degradation of polyesters ^14,15,18^, were also more enriched in the PBAT-exposed secretome (Figure 3a), having abundances nearly 3-fold higher than in the medium not added with PBAT.

Differential abundance of proteins involved in response to chemicals was also observed. Muramidase (A0A179G0Y3_PURLI); murein transglycosylase (W9Y456_9EURO) and Cathepsin D (H6BU59_EXODN) were present in the PBAT-exposed secretomes at levels over 20-, and over 5-fold, respectively than those in the unexposed samples. Whereas muramidases and transglycosylases are extracellular hydrolases responsible for microbial degradation by cleavage at the peptidoglycan level ^56,57^, the lysosomal proteases cathepsins possess highly specific proteolytic activity and can be secreted under different kinds of stress ^58^. PBAT additionally triggered the upregulation of fungal phosphatases – known to regulate pathways important for stress – and of nucleoside modifying enzymes, specifically of those involved in thio-modifications of tRNA, the latter essential mediators in a large number of stress responses when proper tRNA modification is a determinant for maintenance of cellular function and viability ^59,60^

*K. chersonesos* Mut, unexposed and PBAT-exposed cultivation in minimal medium

The comparison between PBAT-exposed and unexposed condition yielded a total of 27 proteins with increased and 142 proteins with decreased abundance in the exposed secretome of *K. chersonesos* Mut grown in minimal medium. Along with protein modulation, the presence of On/Off proteins detected exclusively at one of the experimental conditions, was recorded: Whereas 23 upregulated proteins were only observed upon incubation with PBAT, 112 downregulated proteins were found solely in the unexposed secretome. The ratio of secreted to intracellular proteins was approximately 30%:70% (Supplementary Table S3).

As shown in Supplement. Fig. S5b, protein downregulation was detected as the prevalent reaction to PBAT in the mutant across all the identified enriched GO terms categories. Along with lipid metabolism (35% of GO terms associated to downregulated proteins), protein catabolism (16%), vesicle-mediated transport (10%), and response to stress (9%) collected the highest number of downregulated proteins. Protein modulation affected a number of enzymes involved in carbohydrate degradation. The endochitinase B1 (A0A0N1HE03_9EURO) was present in the exposed samples at level 20-fold lower than that of the unexposed ones. L-xylo-3-hexulose reductase (A0A438MZU1_EXOME), beta-glucosidase (W9YAZ0_9EURO), endoglucanase (H6C6J4_EXODN), glycoside hydrolase family 61 (A0A0G2FDQ5_9PEZI) and TIGR01456 family HAD hydrolase (A0A0D1X661_9EURO) were uniquely detected in the control secretome. Conversely, increased levels were observed for glycoside hydrolase family 16 (A0A1C1D0J4_9EURO; levels 37-fold higher than in the unexposed secretomes) and family 28 (A0A1Y2VIA8_9PEZI), the latter only detected in the exposed samples. In a similar fashion, esterolytic enzymes – i.e. cutinase and COesterase – could be found exclusively upon PBAT incubation (Supplementary Table S4). Regulation of proteins involved in protein catabolism and stress response was also observed. The intracellular SSD1 (A0A0N1HLI9_9EURO) – reported to play a regulatory role in polar growth and wall integrity ^61^ – fibrillarin (W9Z6G9_9EURO), peptidyl-prolyl cis-trans isomerase (A0A0D2BRZ4_9EURO) involved in protein folding, translation initiation factor (V9DRM7_9EURO) and cysteinyl-tRNA synthetase (W9XT49_9EURO) involved in the transcription and translation process ^62^, were only present in the PBAT-exposed samples. Decreased levels of structural protein constituent of ribosome were instead observed in secretomes exposed to PBAT. A number of additional proteins enriched in the unexposed secretomes, whose identity or function could not be elucidated also after homology search, are not displayed in Supplementary Table S4.

*K. chersonesos* Wt and Mut exhibit opposite responses to PBAT at the secretome level

One characteristic of *K. chersonesos* Wt was increased abundance of a number of carbohydrate-active enzymes such as glucanase and endo-acting mannosidases, along with the aforementioned carboxylic ester hydrolases. This is consistent with previous reports of enhanced metabolism in black yeasts and aromatic compounds degraders when grown in presence of polyaromatic hydrocarbons ^35^. The PBAT-dependent increase in the levels of proteins known to play a role in the degradation of complex carbohydrates ^71^ might thereby support *K. chersonesos* oligotrophic nature. The ability of the Wt to degrade PBAT, however goes hand in hand with the upregulation of chemical stress component proteins with proteolytic and phosphatase activity which might indicate that the wild type senses the polymer. In contrast to the Wt, the mutant’s response to PBAT was protein downregulation (Figure S5b), which suggests a general slowing of the metabolic rate. The recourse to an energy-saving state under suboptimal growth conditions is indeed in line with what previously reported in black fungi ^26–28,35,72^. In the Mut more than in the Wt, a substantial number of On/Off proteins were detected exclusively upon exposure to PBAT (Table S4). These proteins included enzymes involved in protein folding as well as carbohydrate-active enzymes, the latter present in the mutant secretome at a much lower levels than in the wild type. Moreover, around 70% of the modulated proteins encompassed intracellular proteins, which can be interpreted as a hallmark of cell lysis (Table S3).

**Supplementary Table S3**

**Supplementary Table S3_** Predicted sub-cellular localizations of the proteins regulated in the secretome of *K. chersonesos* Wt and Mut during cultivation in minimal medium in presence of PBAT, as compared to the unexposed samples.

| ***Knufia chersonesos,* Wt** | | | | | | | | | | |  |
| --- | --- | --- | --- | --- | --- | --- | --- | --- | --- | --- | --- |
| **Protein Accession No. ^a^** | | **GO-ID** | | **GO-term** | | **Score** | | **Alternative Localization ^b^** | | **Features** |  |
| **Up-regulated proteins** (unique to the PBAT-exposed secretomes) | | | | | | | | | | |  |
| g7863.t1 | | [GO:0012505](https://www.ebi.ac.uk/QuickGO/term/GO:0012505) | | C:endomembrane system | | 0.43 | | [GO:0005886 - C:plasma membrane (score=0.16)](https://www.ebi.ac.uk/QuickGO/term/GO:0005886) | | Signal Peptide, Transmembrane Alpha Helix |  |
| g7511.t1 | | [GO:0005615](https://www.ebi.ac.uk/QuickGO/term/GO:0005615) | | C:extracellular space | | 0.8 | | - | | Signal Peptide |  |
| g818.t1 | | [GO:0005886](https://www.ebi.ac.uk/QuickGO/term/GO:0005886) | | C:plasma membrane | | 0.92 | | [GO:0031090 - C:organelle membrane (score=0.67)](https://www.ebi.ac.uk/QuickGO/term/GO:0031090) | | Transmembrane Alpha Helix |  |
| **Up-regulated proteins** | | | | | | | | | | |  |
| g5620.t1 | | [GO:0005615](https://www.ebi.ac.uk/QuickGO/term/GO:0005615) | | C:extracellular space | | 0.99 | | - | | Signal Peptide |  |
| g4716.t1 | | [GO:0005615](https://www.ebi.ac.uk/QuickGO/term/GO:0005615) | | C:extracellular space | | 0.79 | | - | | Signal Peptide |  |
| 7972.t1 | | [GO:0005615](https://www.ebi.ac.uk/QuickGO/term/GO:0005615) | | C:extracellular space | | 0.98 | | - | | Signal Peptide |  |
| g1521.t1 | | [GO:0046658](https://www.ebi.ac.uk/QuickGO/term/GO:0046658) | | C:anchored component of plasma membrane | | 0.99 | | - | | Signal Peptide,GPI-anchor |  |
| g4652.t1 | | [GO:0005739](https://www.ebi.ac.uk/QuickGO/term/GO:0005739) | | C:mitochondrion | | 0.53 | | - | | Mitochondrial Transit Peptide |  |
| g3149.t1 | | [GO:0046658](https://www.ebi.ac.uk/QuickGO/term/GO:0046658) | | C:anchored component of plasma membrane | | 1 | | - | | Signal Peptide, GPI-anchor |  |
| g3426.t1 | | [GO:0005615](https://www.ebi.ac.uk/QuickGO/term/GO:0005615) | | C:extracellular space | | 0.98 | | - | | Signal Peptide |  |
| g4930.t1 | | [GO:0005615](https://www.ebi.ac.uk/QuickGO/term/GO:0005615) | | C:extracellular space | | 0.94 | | - | | Signal Peptide |  |
| g7540.t1 | | [GO:0005615](https://www.ebi.ac.uk/QuickGO/term/GO:0005615) | | C:extracellular space | | 0.94 | | - | | Signal Peptide |  |
| g1769.t1 | | [GO:0005615](https://www.ebi.ac.uk/QuickGO/term/GO:0005615) | | C:extracellular space | | 1 | | - | |  |  |
| g4842.t1 | | [GO:0005615](https://www.ebi.ac.uk/QuickGO/term/GO:0005615) | | C:extracellular space | | 0.98 | | - | | Signal Peptide |  |
| g2946.t1 | | [GO:0005615](https://www.ebi.ac.uk/QuickGO/term/GO:0005615) | | C:extracellular space | | 0.97 | | - | | Signal Peptide |  |
| g6630.t1 | | [GO:0046658](https://www.ebi.ac.uk/QuickGO/term/GO:0046658) | | C:anchored component of plasma membrane | | 0.99 | | - | | Signal Peptide,GPI-anchor |  |
| g8540.t1 | | [GO:0046658](https://www.ebi.ac.uk/QuickGO/term/GO:0046658) | | C:anchored component of plasma membrane | | 0.99 | | - | | Signal Peptide,GPI-anchor |  |
| g6975.t1 | | [GO:0005886](https://www.ebi.ac.uk/QuickGO/term/GO:0005886) | | C:plasma membrane | | 0.82 | | - | | Signal Peptide,Transmembrane Alpha Helix |  |
| g3613.t1 | | [GO:0005615](https://www.ebi.ac.uk/QuickGO/term/GO:0005615) | | C:extracellular space | | 0.99 | | - | | Signal Peptide |  |
| g8952.t1 | | [GO:0005886](https://www.ebi.ac.uk/QuickGO/term/GO:0005886) | | C:plasma membrane | | 0.92 | | [GO:0012505 - C:endomembrane system (score=0.38)](https://www.ebi.ac.uk/QuickGO/term/GO:0012505) | | Signal Peptide,Transmembrane Alpha Helix |  |
| g1355.t1 | | [GO:0005615](https://www.ebi.ac.uk/QuickGO/term/GO:0005615) | | C:extracellular space | | 1 | | - | | Signal Peptide |  |
| g5159.t1 | | [GO:0005615](https://www.ebi.ac.uk/QuickGO/term/GO:0005615) | | C:extracellular space | | 1 | | - | | Signal Peptide |  |
| g3719.t1 | | [GO:0012505](https://www.ebi.ac.uk/QuickGO/term/GO:0012505) | | C:endomembrane system | | 0.81 | | [GO:0005886 - C:plasma membrane (score=0.66)](https://www.ebi.ac.uk/QuickGO/term/GO:0005886) | | Transmembrane Alpha Helix |  |
| g7098.t1 | | [GO:0046658](https://www.ebi.ac.uk/QuickGO/term/GO:0046658) | | C:anchored component of plasma membrane | | 0.99 | | - | | Signal Peptide,GPI-anchor |  |
| g488.t1 | | [GO:0005615](https://www.ebi.ac.uk/QuickGO/term/GO:0005615) | | C:extracellular space | | 1 | | - | |  |  |
| g7971.t1 | | [GO:0005615](https://www.ebi.ac.uk/QuickGO/term/GO:0005615) | | C:extracellular space | | 0.99 | | - | | Signal Peptide |  |
| g2377.t1 | | [GO:0005615](https://www.ebi.ac.uk/QuickGO/term/GO:0005615) | | C:extracellular space | | 0.78 | | - | | Signal Peptide |  |
| g8605.t1 | | [GO:0005615](https://www.ebi.ac.uk/QuickGO/term/GO:0005615) | | C:extracellular space | | 0.99 | | - | | Signal Peptide |  |
| g4208.t1 | | [GO:0005615](https://www.ebi.ac.uk/QuickGO/term/GO:0005615) | | C:extracellular space | | 0.94 | | - | | Signal Peptide |  |
| g5579.t1 | | [GO:0005615](https://www.ebi.ac.uk/QuickGO/term/GO:0005615) | | C:extracellular space | | 0.92 | | - | | Signal Peptide |  |
| g1975.t1 | | [GO:0005615](https://www.ebi.ac.uk/QuickGO/term/GO:0005615) | | C:extracellular space | | 1 | | - | | Signal Peptide |  |
| g6880.t1 | | [GO:0005615](https://www.ebi.ac.uk/QuickGO/term/GO:0005615) | | C:extracellular space | | 1 | | - | |  |  |
| g6247.t1 | | [GO:0046658](https://www.ebi.ac.uk/QuickGO/term/GO:0046658) | | C:anchored component of plasma membrane | | 0.98 | | - | | Signal Peptide,GPI-anchor |  |
| g7566.t1 | | [GO:0005615](https://www.ebi.ac.uk/QuickGO/term/GO:0005615) | | C:extracellular space | | 1 | | - | | Signal Peptide |  |
| g7569.t1 | | [GO:0005615](https://www.ebi.ac.uk/QuickGO/term/GO:0005615) | | C:extracellular space | | 1 | | - | | Signal Peptide |  |
| g9529.t1 | | [GO:0005615](https://www.ebi.ac.uk/QuickGO/term/GO:0005615) | | C:extracellular space | | 0.98 | | - | | Signal Peptide |  |
| g4908.t1 | | [GO:0005737](https://www.ebi.ac.uk/QuickGO/term/GO:0005737) | | C:cytoplasm | | 0.7 | | [GO:0005634 - C:nucleus (score=0.3)](https://www.ebi.ac.uk/QuickGO/term/GO:0005634) | |  |  |
| g6203.t1 | | [GO:0005886](https://www.ebi.ac.uk/QuickGO/term/GO:0005886) | | C:plasma membrane | | 0.8 | | - | | Signal Peptide,Transmembrane Alpha Helix |  |
| g3601.t1 | | [GO:0012505](https://www.ebi.ac.uk/QuickGO/term/GO:0012505) | | C:endomembrane system | | 0.56 | | - | | Signal Peptide,Transmembrane Alpha Helix |  |
| g3855.t1 | | [GO:0005615](https://www.ebi.ac.uk/QuickGO/term/GO:0005615) | | C:extracellular space | | 0.75 | | - | | Signal Peptide |  |
| g6751.t1 | | [GO:0005615](https://www.ebi.ac.uk/QuickGO/term/GO:0005615) | | C:extracellular space | | 0.96 | | - | | Signal Peptide |  |
| g5038.t1 | | [GO:0046658](https://www.ebi.ac.uk/QuickGO/term/GO:0046658) | | C:anchored component of plasma membrane | | 0.99 | | - | | Signal Peptide,GPI-anchor |  |
| g583.t1 | | [GO:0046658](https://www.ebi.ac.uk/QuickGO/term/GO:0046658) | | C:anchored component of plasma membrane | | 0.99 | | - | | Signal Peptide,GPI-anchor |  |
| g8924.t1 | | [GO:0046658](https://www.ebi.ac.uk/QuickGO/term/GO:0046658) | | C:anchored component of plasma membrane | | 1 | | - | | Signal Peptide,GPI-anchor |  |
| g6769.t1 | | [GO:0005886](https://www.ebi.ac.uk/QuickGO/term/GO:0005886) | | C:plasma membrane | | 0.89 | | [GO:0012505 - C:endomembrane system (score=0.42)](https://www.ebi.ac.uk/QuickGO/term/GO:0012505) | | Transmembrane Alpha Helix |  |
| g6689.t1 | | [GO:0005615](https://www.ebi.ac.uk/QuickGO/term/GO:0005615) | | C:extracellular space | | 1 | | - | | Signal Peptide |  |
| g8444.t1 | | [GO:0005886](https://www.ebi.ac.uk/QuickGO/term/GO:0005886) | | C:plasma membrane | | 0.88 | | [GO:0012505 - C:endomembrane system (score=0.16)](https://www.ebi.ac.uk/QuickGO/term/GO:0012505) | | Signal Peptide,Transmembrane Alpha Helix |  |
| g1867.t1 | | [GO:0005615](https://www.ebi.ac.uk/QuickGO/term/GO:0005615) | | C:extracellular space | | 1 | | - | | Signal Peptide |  |
| g7970.t1 | | [GO:0005886](https://www.ebi.ac.uk/QuickGO/term/GO:0005886) | | C:plasma membrane | | 0.9 | | - | | Transmembrane Alpha Helix |  |
| g2279.t1 | | [GO:0005615](https://www.ebi.ac.uk/QuickGO/term/GO:0005615) | | C:extracellular space | | 0.99 | | - | | Signal Peptide |  |
| g4729.t1 | | [GO:0012505](https://www.ebi.ac.uk/QuickGO/term/GO:0012505) | | C:endomembrane system | | 0.85 | | [GO:0031090 - C:organelle membrane (score=0.6)](https://www.ebi.ac.uk/QuickGO/term/GO:0031090) | | Transmembrane Alpha Helix |  |
| g7683.t1 | | [GO:0005615](https://www.ebi.ac.uk/QuickGO/term/GO:0005615) | | C:extracellular space | | 0.98 | | - | | Signal Peptide |  |
| g8179.t1 | | [GO:0005615](https://www.ebi.ac.uk/QuickGO/term/GO:0005615) | | C:extracellular space | | 1 | | - | |  |  |
| g7208.t1 | | [GO:0005737](https://www.ebi.ac.uk/QuickGO/term/GO:0005737) | | C:cytoplasm | | 1 | | - | |  |  |
| g3227.t1 | | [GO:0005615](https://www.ebi.ac.uk/QuickGO/term/GO:0005615) | | C:extracellular space | | 1 | | - | |  |  |
| g4123.t1 | | [GO:0005886](https://www.ebi.ac.uk/QuickGO/term/GO:0005886) | | C:plasma membrane | | 0.82 | | - | | Signal Peptide,Transmembrane Alpha Helix |  |
| g8836.t1 | | [GO:0005615](https://www.ebi.ac.uk/QuickGO/term/GO:0005615) | | C:extracellular space | | 0.93 | | - | | Signal Peptide |  |
| g3415.t1 | | [GO:0005886](https://www.ebi.ac.uk/QuickGO/term/GO:0005886) | | C:plasma membrane | | 0.89 | | [GO:0031090 - C:organelle membrane (score=0.42)](https://www.ebi.ac.uk/QuickGO/term/GO:0031090) | | Transmembrane Alpha Helix |  |
| g4369.t1 | | [GO:0005737](https://www.ebi.ac.uk/QuickGO/term/GO:0005737) | | C:cytoplasm | | 0.82 | | [GO:0005634 - C:nucleus (score=0.18)](https://www.ebi.ac.uk/QuickGO/term/GO:0005634) | |  |  |
| g3607.t1 | | [GO:0005615](https://www.ebi.ac.uk/QuickGO/term/GO:0005615) | | C:extracellular space | | 1 | | - | | Signal Peptide |  |
| g1458.t1 | | [GO:0046658](https://www.ebi.ac.uk/QuickGO/term/GO:0046658) | | C:anchored component of plasma membrane | | 0.99 | | - | | Signal Peptide,GPI-anchor |  |
| g8229.t1 | | [GO:0046658](https://www.ebi.ac.uk/QuickGO/term/GO:0046658) | | C:anchored component of plasma membrane | | 1 | | - | | Signal Peptide,GPI-anchor |  |
| g4251.t1 | | [GO:0005615](https://www.ebi.ac.uk/QuickGO/term/GO:0005615) | | C:extracellular space | | 0.98 | | - | | Signal Peptide |  |
| g3201.t1 | | [GO:0005615](https://www.ebi.ac.uk/QuickGO/term/GO:0005615) | | C:extracellular space | | 0.84 | | - | | Signal Peptide |  |
| g1109.t1 | | [GO:0005615](https://www.ebi.ac.uk/QuickGO/term/GO:0005615) | | C:extracellular space | | 1 | | - | | Signal Peptide |  |
| g7701.t1 | | [GO:0005615](https://www.ebi.ac.uk/QuickGO/term/GO:0005615) | | C:extracellular space | | 0.97 | | - | | Signal Peptide |  |
| g2826.t1 | | [GO:0005615](https://www.ebi.ac.uk/QuickGO/term/GO:0005615) | | C:extracellular space | | 0.99 | | - | | Signal Peptide |  |
| **Down-regulated proteins** | | | | | | | | | | |  |
| g8214.t1 | | [GO:0005737](https://www.ebi.ac.uk/QuickGO/term/GO:0005737) | | C:cytoplasm | | 0.7 | | [GO:0005634 - C:nucleus (score=0.3)](https://www.ebi.ac.uk/QuickGO/term/GO:0005634) | |  |  |
| g8842.t1 | | [GO:0005737](https://www.ebi.ac.uk/QuickGO/term/GO:0005737) | | C:cytoplasm | | 0.7 | | [GO:0005634 - C:nucleus (score=0.3)](https://www.ebi.ac.uk/QuickGO/term/GO:0005634) | |  |  |
| g1243.t1 | | [GO:0005615](https://www.ebi.ac.uk/QuickGO/term/GO:0005615) | | C:extracellular space | | 1 | | - | |  |  |
| g7256.t1 | | [GO:0005739](https://www.ebi.ac.uk/QuickGO/term/GO:0005739) | | C:mitochondrion | | 1 | | - | |  |  |
| g8648.t1 | | [GO:0005739](https://www.ebi.ac.uk/QuickGO/term/GO:0005739) | | C:mitochondrion | | 0.98 | | - | | Mitochondrial Transit Peptide |  |
| g2250.t1 | | [GO:0005737](https://www.ebi.ac.uk/QuickGO/term/GO:0005737) | | C:cytoplasm | | 1 | | - | |  |  |
| g2047.t1 | | [GO:0005634](https://www.ebi.ac.uk/QuickGO/term/GO:0005634) | | C:nucleus | | 1 | | - | |  |  |
| g6990.t1 | | [GO:0005737](https://www.ebi.ac.uk/QuickGO/term/GO:0005737) | | C:cytoplasm | | 0.7 | | [GO:0005634 - C:nucleus (score=0.3)](https://www.ebi.ac.uk/QuickGO/term/GO:0005634) | |  |  |
| g1779.t1 | | [GO:0005615](https://www.ebi.ac.uk/QuickGO/term/GO:0005615) | | C:extracellular space | | 0.91 | | - | | Signal Peptide |  |
| g3435.t1 | | [GO:0005615](https://www.ebi.ac.uk/QuickGO/term/GO:0005615) | | C:extracellular space | | 0.99 | | - | | Signal Peptide |  |
| g1815.t1 | | [GO:0005737](https://www.ebi.ac.uk/QuickGO/term/GO:0005737) | | C:cytoplasm | | 1 | | - | |  |  |
| g1888.t1 | | [GO:0005634](https://www.ebi.ac.uk/QuickGO/term/GO:0005634) | | C:nucleus | | 1 | | - | |  |  |
| g3234.t1 | | [GO:0005615](https://www.ebi.ac.uk/QuickGO/term/GO:0005615) | | C:extracellular space | | 0.99 | | - | | Signal Peptide |  |
| g4993.t1 | | [GO:0005634](https://www.ebi.ac.uk/QuickGO/term/GO:0005634) | | C:nucleus | | 1 | | - | |  |  |
| g567.t1 | | [GO:0005737](https://www.ebi.ac.uk/QuickGO/term/GO:0005737) | | C:cytoplasm | | 0.7 | | [GO:0005634 - C:nucleus (score=0.3)](https://www.ebi.ac.uk/QuickGO/term/GO:0005634) | |  |  |
| g4686.t1 | | [GO:0005737](https://www.ebi.ac.uk/QuickGO/term/GO:0005737) | | C:cytoplasm | | 1 | | - | |  |  |
| g3711.t1 | | [GO:0005737](https://www.ebi.ac.uk/QuickGO/term/GO:0005737) | | C:cytoplasm | | 0.7 | | [GO:0005634 - C:nucleus (score=0.3)](https://www.ebi.ac.uk/QuickGO/term/GO:0005634) | |  |  |
| g1443.t1 | | [GO:0005615](https://www.ebi.ac.uk/QuickGO/term/GO:0005615) | | C:extracellular space | | 0.97 | | - | | Signal Peptide |  |
| g8393.t1 | | [GO:0005737](https://www.ebi.ac.uk/QuickGO/term/GO:0005737) | | C:cytoplasm | | 0.7 | | [GO:0005634 - C:nucleus (score=0.3)](https://www.ebi.ac.uk/QuickGO/term/GO:0005634) | |  |  |
| g4660.t1 | | [GO:0005615](https://www.ebi.ac.uk/QuickGO/term/GO:0005615) | | C:extracellular space | | 0.77 | | - | | Signal Peptide |  |
| g5874.t1 | | [GO:0005737](https://www.ebi.ac.uk/QuickGO/term/GO:0005737) | | C:cytoplasm | | 0.7 | | [GO:0005634 - C:nucleus (score=0.3)](https://www.ebi.ac.uk/QuickGO/term/GO:0005634) | |  |  |
| g5495.t1 | | [GO:0005615](https://www.ebi.ac.uk/QuickGO/term/GO:0005615) | | C:extracellular space | | 0.9 | | - | | Signal Peptide |  |
| g1827.t1 | | [GO:0005737](https://www.ebi.ac.uk/QuickGO/term/GO:0005737) | | C:cytoplasm | | 1 | | - | |  |  |
| g100.t1 | | [GO:0005615](https://www.ebi.ac.uk/QuickGO/term/GO:0005615) | | C:extracellular space | | 0.97 | | - | | Signal Peptide |  |
| g6301.t1 | | [GO:0005615](https://www.ebi.ac.uk/QuickGO/term/GO:0005615) | | C:extracellular space | | 0.96 | | - | | Signal Peptide |  |
| g7081.t1 | | [GO:0005615](https://www.ebi.ac.uk/QuickGO/term/GO:0005615) | | C:extracellular space | | 1 | | - | | Signal Peptide |  |
| g9126.t1 | | [GO:0005615](https://www.ebi.ac.uk/QuickGO/term/GO:0005615) | | C:extracellular space | | 1 | | - | | Signal Peptide |  |
| g6063.t1 | | [GO:0005737](https://www.ebi.ac.uk/QuickGO/term/GO:0005737) | | C:cytoplasm | | 1 | | - | |  |  |
| g7438.t1 | | [GO:0005634](https://www.ebi.ac.uk/QuickGO/term/GO:0005634) | | C:nucleus | | 1 | | - | |  |  |
| g5735.t1 | | [GO:0005634](https://www.ebi.ac.uk/QuickGO/term/GO:0005634) | | C:nucleus | | 1 | | - | |  |  |
| g7536.t1 | | [GO:0005615](https://www.ebi.ac.uk/QuickGO/term/GO:0005615) | | C:extracellular space | | 1 | | - | |  |  |
| g389.t1 | | [GO:0005615](https://www.ebi.ac.uk/QuickGO/term/GO:0005615) | | C:extracellular space | | 1 | | - | |  |  |
| g3679.t1 | | [GO:0005615](https://www.ebi.ac.uk/QuickGO/term/GO:0005615) | | C:extracellular space | | 1 | | - | |  |  |
| g9210.t1 | | [GO:0005737](https://www.ebi.ac.uk/QuickGO/term/GO:0005737) | | C:cytoplasm | | 0.7 | | [GO:0005634 - C:nucleus (score=0.3)](https://www.ebi.ac.uk/QuickGO/term/GO:0005634) | |  |  |
| g6530.t1 | | [GO:0005737](https://www.ebi.ac.uk/QuickGO/term/GO:0005737) | | C:cytoplasm | | 0.7 | | [GO:0005634 - C:nucleus (score=0.3)](https://www.ebi.ac.uk/QuickGO/term/GO:0005634) | |  |  |
| **Down-regulated proteins** (unique to the unexposed secretomes) | | | | | | | | | | |  |
| g9343.t1 | | [GO:0012505](https://www.ebi.ac.uk/QuickGO/term/GO:0012505) | | C:endomembrane system | | 0.9 | | - | | Signal Peptide,Transmembrane Alpha Helix |  |
| g6347.t1 | | [GO:0005634](https://www.ebi.ac.uk/QuickGO/term/GO:0005634) | | C:nucleus | | 1 | | - | |  |  |
| g2825.t1 | | [GO:0005737](https://www.ebi.ac.uk/QuickGO/term/GO:0005737) | | C:cytoplasm | | 1 | | - | |  |  |
| g1432.t1 | | [GO:0005737](https://www.ebi.ac.uk/QuickGO/term/GO:0005737) | | C:cytoplasm | | 0.7 | | [GO:0005634 - C:nucleus (score=0.3)](https://www.ebi.ac.uk/QuickGO/term/GO:0005634) | |  |  |
| g7744.t1 | | [GO:0005886](https://www.ebi.ac.uk/QuickGO/term/GO:0005886) | | C:plasma membrane | | 0.82 | | [GO:0012505 - C:endomembrane system (score=0.29)](https://www.ebi.ac.uk/QuickGO/term/GO:0012505) | | Transmembrane Alpha Helix |  |
| g5512.t1 | | [GO:0005615](https://www.ebi.ac.uk/QuickGO/term/GO:0005615) | | C:extracellular space | | 0.89 | | - | | Signal Peptide |  |
| g1226.t1 | | [GO:0005615](https://www.ebi.ac.uk/QuickGO/term/GO:0005615) | | C:extracellular space | | 0.99 | | - | | Signal Peptide |  |
| g9764.t1 | | [GO:0005615](https://www.ebi.ac.uk/QuickGO/term/GO:0005615) | | C:extracellular space | | 1 | | - | | Signal Peptide |  |
| g362.t1 | | [GO:0005615](https://www.ebi.ac.uk/QuickGO/term/GO:0005615) | | C:extracellular space | | 1 | | - | |  |  |
| g4790.t1 | | [GO:0005737](https://www.ebi.ac.uk/QuickGO/term/GO:0005737) | | C:cytoplasm | | 0.7 | | [GO:0005634 - C:nucleus (score=0.3)](https://www.ebi.ac.uk/QuickGO/term/GO:0005634) | |  |  |
| g5543.t1 | | [GO:0005737](https://www.ebi.ac.uk/QuickGO/term/GO:0005737) | | C:cytoplasm | | 0.7 | | [GO:0005634 - C:nucleus (score=0.3)](https://www.ebi.ac.uk/QuickGO/term/GO:0005634) | |  |  |
| g2351.t1 | | [GO:0005737](https://www.ebi.ac.uk/QuickGO/term/GO:0005737) | | C:cytoplasm | | 1 | | - | |  |  |
| g7238.t1 | | [GO:0005615](https://www.ebi.ac.uk/QuickGO/term/GO:0005615) | | C:extracellular space | | 1 | | - | |  |  |
| g8255.t1 | | [GO:0005737](https://www.ebi.ac.uk/QuickGO/term/GO:0005737) | | C:cytoplasm | | 1 | | - | |  |  |
| g1417.t1 | | [GO:0005615](https://www.ebi.ac.uk/QuickGO/term/GO:0005615) | | C:extracellular space | | 1 | | - | |  |  |
| g1930.t1 | | [GO:0005739](https://www.ebi.ac.uk/QuickGO/term/GO:0005739) | | C:mitochondrion | | 1 | | - | |  |  |
| g6772.t1 | | [GO:0005739](https://www.ebi.ac.uk/QuickGO/term/GO:0005739) | | C:mitochondrion | | 0.74 | | - | | Mitochondrial Transit Peptide |  |
| g3627.t1 | | [GO:0005634](https://www.ebi.ac.uk/QuickGO/term/GO:0005634) | | C:nucleus | | 1 | | - | |  |  |
| g1227.t1 | | [GO:0005615](https://www.ebi.ac.uk/QuickGO/term/GO:0005615) | | C:extracellular space | | 0.99 | | - | | Signal Peptide |  |
| g689.t1 | | [GO:0005615](https://www.ebi.ac.uk/QuickGO/term/GO:0005615) | | C:extracellular space | | 0.93 | | - | | Signal Peptide |  |
| ***Knufia chersonesos,* Mut** | | | | | | | | | | |  |
| **Protein Accession/ID** | **GO-id** | | **GO-term** | | **Score** | | **Alternative Localization** | | **Features** | |  |
| **Up-regulated proteins** (unique to the PBAT-exposed secretomes) | | | | | | | | | | |  |
| g4295.t1 | [GO:0005615](https://www.ebi.ac.uk/QuickGO/term/GO:0005615) | | C:extracellular space | | 1 | | - | | Signal Peptide | |  |
| g2467.t1 | [GO:0005737](https://www.ebi.ac.uk/QuickGO/term/GO:0005737) | | C:cytoplasm | | 1 | | - | |  | |  |
| g7390.t1 | [GO:0005737](https://www.ebi.ac.uk/QuickGO/term/GO:0005737) | | C:cytoplasm | | 0.7 | | [GO:0005634 - C:nucleus (score=0.3)](https://www.ebi.ac.uk/QuickGO/term/GO:0005634) | |  | |  |
| g4619.t1 | [GO:0005615](https://www.ebi.ac.uk/QuickGO/term/GO:0005615) | | C:extracellular space | | 1 | | - | | Signal Peptide | |  |
| g1282.t1 | | [GO:0005737](https://www.ebi.ac.uk/QuickGO/term/GO:0005737) | | C:cytoplasm | | 0.7 | | [GO:0005634 - C:nucleus (score=0.3)](https://www.ebi.ac.uk/QuickGO/term/GO:0005634) | |  | |
| g4772.t1 | | [GO:0005634](https://www.ebi.ac.uk/QuickGO/term/GO:0005634) | | C:nucleus | | 1 | | - | |  | |
| g978.t1 | | [GO:0005737](https://www.ebi.ac.uk/QuickGO/term/GO:0005737) | | C:cytoplasm | | 0.7 | | [GO:0005634 - C:nucleus (score=0.3)](https://www.ebi.ac.uk/QuickGO/term/GO:0005634) | |  | |
| g943.t1 | | [GO:0005737](https://www.ebi.ac.uk/QuickGO/term/GO:0005737) | | C:cytoplasm | | 0.52 | | [GO:0005634 - C:nucleus (score=0.48)](https://www.ebi.ac.uk/QuickGO/term/GO:0005634) | |  | |
| g4392.t1 | | [GO:0005634](https://www.ebi.ac.uk/QuickGO/term/GO:0005634) | | C:nucleus | | 1 | | - | |  | |
| g2805.t1 | | [GO:0005634](https://www.ebi.ac.uk/QuickGO/term/GO:0005634) | | C:nucleus | | 1 | | - | |  | |
| g8915.t1 | | [GO:0046658](https://www.ebi.ac.uk/QuickGO/term/GO:0046658) | | C:anchored component of plasma membrane | | 1 | | - | |  | |
| g3515.t1 | | [GO:0005634](https://www.ebi.ac.uk/QuickGO/term/GO:0005634) | | C:nucleus | | 1 | | - | |  | |
| g4767.t1 | | [GO:0005739](https://www.ebi.ac.uk/QuickGO/term/GO:0005739) | | C:mitochondrion | | 0.84 | | - | | Mitochondrial Transit Peptide | |
| g5720.t1 | | [GO:0005634](https://www.ebi.ac.uk/QuickGO/term/GO:0005634) | | C:nucleus | | 1 | | - | |  | |
| g8970.t1 | | [GO:0005737](https://www.ebi.ac.uk/QuickGO/term/GO:0005737) | | C:cytoplasm | | 1 | | - | |  | |
| g7633.t1 | | [GO:0005737](https://www.ebi.ac.uk/QuickGO/term/GO:0005737) | | C:cytoplasm | | 1 | | - | |  | |
| g818.t1 | | [GO:0005886](https://www.ebi.ac.uk/QuickGO/term/GO:0005886) | | C:plasma membrane | | 0.92 | | [GO:0031090 - C:organelle membrane (score=0.67)](https://www.ebi.ac.uk/QuickGO/term/GO:0031090) | | Transmembrane Alpha Helix | |
| g395.t1 | | [GO:0005737](https://www.ebi.ac.uk/QuickGO/term/GO:0005737) | | C:cytoplasm | | 1 | | - | |  | |
| g315.t1 | | [GO:0005615](https://www.ebi.ac.uk/QuickGO/term/GO:0005615) | | C:extracellular space | | 1 | | - | |  | |
| g6908.t1 | | [GO:0005615](https://www.ebi.ac.uk/QuickGO/term/GO:0005615) | | C:extracellular space | | 0.87 | | [GO:0005634 - C:nucleus (score=0.13)](https://www.ebi.ac.uk/QuickGO/term/GO:0005634) | |  | |
| g4805.t1 | | [GO:0005737](https://www.ebi.ac.uk/QuickGO/term/GO:0005737) | | C:cytoplasm | | 0.57 | | [GO:0005634 - C:nucleus (score=0.43)](https://www.ebi.ac.uk/QuickGO/term/GO:0005634) | |  | |
| g304.t1 | | [GO:0005737](https://www.ebi.ac.uk/QuickGO/term/GO:0005737) | | C:cytoplasm | | 1 | | - | |  | |
| **Up-regulated proteins** | | | | | | | | | | | |
| g8229.t1 | | [GO:0046658](https://www.ebi.ac.uk/QuickGO/term/GO:0046658) | | C:anchored component of plasma membrane | | 1 | | - | | Signal Peptide,GPI-anchor | |
| g7863.t1 | | [GO:0012505](https://www.ebi.ac.uk/QuickGO/term/GO:0012505) | | C:endomembrane system | | 0.43 | | [GO:0005886 - C:plasma membrane (score=0.16)](https://www.ebi.ac.uk/QuickGO/term/GO:0005886) | | Signal Peptide,Transmembrane Alpha Helix | |
| g3149.t1 | | [GO:0046658](https://www.ebi.ac.uk/QuickGO/term/GO:0046658) | | C:anchored component of plasma membrane | | 1 | | - | | Signal Peptide,GPI-anchor | |
| g3426.t1 | | [GO:0005615](https://www.ebi.ac.uk/QuickGO/term/GO:0005615) | | C:extracellular space | | 0.98 | | - | | Signal Peptide | |
| g4716.t1 | | [GO:0005615](https://www.ebi.ac.uk/QuickGO/term/GO:0005615) | | C:extracellular space | | 0:79 | | - | | Signal Peptide | |
| **Down-regulated proteins** | | | | | | | | | | | |
| g5324.t1 | | [GO:0005886](https://www.ebi.ac.uk/QuickGO/term/GO:0005886) | | C:plasma membrane | | 0.85 | | - | | Transmembrane Alpha Helix | |
| g742.t1 | | [GO:0005737](https://www.ebi.ac.uk/QuickGO/term/GO:0005737) | | C:cytoplasm | | 1 | | - | |  | |
| g5017.t1 | | [GO:0005737](https://www.ebi.ac.uk/QuickGO/term/GO:0005737) | | C:cytoplasm | | 0.7 | | [GO:0005634 - C:nucleus (score=0.3)](https://www.ebi.ac.uk/QuickGO/term/GO:0005634) | |  | |
| g2972.t1 | | [GO:0005615](https://www.ebi.ac.uk/QuickGO/term/GO:0005615) | | C:extracellular space | | 1 | | - | |  | |
| g1498.t1 | | [GO:0005737](https://www.ebi.ac.uk/QuickGO/term/GO:0005737) | | C:cytoplasm | | 0.7 | | [GO:0005634 - C:nucleus (score=0.3)](https://www.ebi.ac.uk/QuickGO/term/GO:0005634) | |  | |
| g8443.t1 | | [GO:0005737](https://www.ebi.ac.uk/QuickGO/term/GO:0005737) | | C:cytoplasm | | **1** | | - | |  | |
| g5667.t1 | | [GO:0005739](https://www.ebi.ac.uk/QuickGO/term/GO:0005739) | | C:mitochondrion | | **1** | | - | |  | |
| g314.t1 | | [GO:0005737](https://www.ebi.ac.uk/QuickGO/term/GO:0005737) | | C:cytoplasm | | **1** | | - | |  | |
| g550.t1 | | [GO:0005737](https://www.ebi.ac.uk/QuickGO/term/GO:0005737) | | C:cytoplasm | | **1** | | - | |  | |
| g2669.t1 | | [GO:0005739](https://www.ebi.ac.uk/QuickGO/term/GO:0005739) | | C:mitochondrion | | 0.93 | | - | | Mitochondrial Transit Peptide | |
| g2776.t1 | | [GO:0005737](https://www.ebi.ac.uk/QuickGO/term/GO:0005737) | | C:cytoplasm | | 0.7 | | [GO:0005634 - C:nucleus (score=0.3)](https://www.ebi.ac.uk/QuickGO/term/GO:0005634) | |  | |
| g1405.t1 | | [GO:0005737](https://www.ebi.ac.uk/QuickGO/term/GO:0005737) | | C:cytoplasm | | **1** | | - | |  | |
| g265.t1 | | [GO:0005737](https://www.ebi.ac.uk/QuickGO/term/GO:0005737) | | C:cytoplasm | | **1** | | - | |  | |
| g4711.t1 | | [GO:0005737](https://www.ebi.ac.uk/QuickGO/term/GO:0005737) | | C:cytoplasm | | **0.96** | | - | |  | |
| g7443.t1 | | [GO:0005615](https://www.ebi.ac.uk/QuickGO/term/GO:0005615) | | C:extracellular space | | 0.99 | | - | | Signal Peptide | |
| g7750.t1 | | [GO:0005737](https://www.ebi.ac.uk/QuickGO/term/GO:0005737) | | C:cytoplasm | | 0.7 | | [GO:0005634 - C:nucleus (score=0.3)](https://www.ebi.ac.uk/QuickGO/term/GO:0005634) | | Signal Peptide | |
| g8085.t1 | | [GO:0005615](https://www.ebi.ac.uk/QuickGO/term/GO:0005615) | | C:extracellular space | | 1 | | - | | Signal Peptide | |
| g3234.t1 | | [GO:0005615](https://www.ebi.ac.uk/QuickGO/term/GO:0005615) | | C:extracellular space | | 0.99 | | - | |  | |
| g6685.t1 | | [GO:0005615](https://www.ebi.ac.uk/QuickGO/term/GO:0005615) | | C:extracellular space | | 1 | | - | |  | |
| g2708.t1 | | [GO:0005615](https://www.ebi.ac.uk/QuickGO/term/GO:0005615) | | C:extracellular space | | 1 | | - | | Signal Peptide | |
| g1605.t1 | | [GO:0005634](https://www.ebi.ac.uk/QuickGO/term/GO:0005634) | | C:nucleus | | 1 | | - | |  | |
| g4686.t1 | | [GO:0005737](https://www.ebi.ac.uk/QuickGO/term/GO:0005737) | | C:cytoplasm | | 1 | | - | |  | |
| g9668.t1 | | [GO:0005615](https://www.ebi.ac.uk/QuickGO/term/GO:0005615) | | C:extracellular space | | 0.86 | | - | | Signal Peptide | |
| g8237.t1 | | [GO:0005615](https://www.ebi.ac.uk/QuickGO/term/GO:0005615) | | C:extracellular space | | 0.99 | | - | | Signal Peptide | |
| g2465.t1 | | [GO:0031090](https://www.ebi.ac.uk/QuickGO/term/GO:0031090) | | C:organelle membrane | | 0.65 | | [GO:0012505 - C:endomembrane system (score=0.61)](https://www.ebi.ac.uk/QuickGO/term/GO:0012505) | | Transmembrane Alpha Helix | |
| g1468.t1 | | [GO:0005615](https://www.ebi.ac.uk/QuickGO/term/GO:0005615) | | C:extracellular space | | 1 | | - | | Signal Peptide | |
| g9210.t1 | | [GO:0005737](https://www.ebi.ac.uk/QuickGO/term/GO:0005737) | | C:cytoplasm | | 0.7 | | [GO:0005634 - C:nucleus (score=0.3)](https://www.ebi.ac.uk/QuickGO/term/GO:0005634) | |  | |
| g6074.t1 | | [GO:0005737](https://www.ebi.ac.uk/QuickGO/term/GO:0005737) | | C:cytoplasm | | 1 | | - | |  | |
| g2832.t1 | | [GO:0046658](https://www.ebi.ac.uk/QuickGO/term/GO:0046658) | | C:anchored component of plasma membrane | | 0.99 | | - | | Signal Peptide,GPI-anchor | |
| g3679.t1 | | [GO:0005615](https://www.ebi.ac.uk/QuickGO/term/GO:0005615) | | C:extracellular space | | 1 | | - | |  | |
| **Down-regulated proteins** (unique to the unexposed secretomes) | | | | | | | | | | | |
| g7588.t1 | | [GO:0005634](https://www.ebi.ac.uk/QuickGO/term/GO:0005634) | | C:nucleus | | 1 | | - | |  | |
| g1059.t1 | | [GO:0005737](https://www.ebi.ac.uk/QuickGO/term/GO:0005737) | | C:cytoplasm | | 1 | | - | |  | |
| g244.t1 | | [GO:0005737](https://www.ebi.ac.uk/QuickGO/term/GO:0005737) | | C:cytoplasm | | 0.7 | | [GO:0005634 - C:nucleus (score=0.3)](https://www.ebi.ac.uk/QuickGO/term/GO:0005634) | |  | |
| g3847.t1 | | [GO:0005737](https://www.ebi.ac.uk/QuickGO/term/GO:0005737) | | C:cytoplasm | | 0.7 | | [GO:0005634 - C:nucleus (score=0.3)](https://www.ebi.ac.uk/QuickGO/term/GO:0005634) | |  | |
| g6026.t1 | | [GO:0005739](https://www.ebi.ac.uk/QuickGO/term/GO:0005739) | | C:mitochondrion | | 1 | |  | |  | |
| g6086.t1 | | [GO:0005737](https://www.ebi.ac.uk/QuickGO/term/GO:0005737) | | C:cytoplasm | | 1 | |  | |  | |
| g1888.t1 | | [GO:0005634](https://www.ebi.ac.uk/QuickGO/term/GO:0005634) | | C:nucleus | | 1 | | - | |  | |
| g5286.t1 | | [GO:0005739](https://www.ebi.ac.uk/QuickGO/term/GO:0005739) | | C:mitochondrion | | 0.76 | | - | | Mitochondrial Transit Peptide | |
| g5094.t1 | | [GO:0005737](https://www.ebi.ac.uk/QuickGO/term/GO:0005737) | | C:cytoplasm | | 1 | | - | |  | |
| g389.t1 | | [GO:0005615](https://www.ebi.ac.uk/QuickGO/term/GO:0005615) | | C:extracellular space | | 1 | | - | |  | |
| g6082.t1 | | [GO:0005737](https://www.ebi.ac.uk/QuickGO/term/GO:0005737) | | C:cytoplasm | | 0.7 | | [GO:0005634 - C:nucleus (score=0.3)](https://www.ebi.ac.uk/QuickGO/term/GO:0005634) | |  | |
| g1432.t1 | | [GO:0005737](https://www.ebi.ac.uk/QuickGO/term/GO:0005737) | | C:cytoplasm | | 0.7 | | [GO:0005634 - C:nucleus (score=0.3)](https://www.ebi.ac.uk/QuickGO/term/GO:0005634) | |  | |
| g7717.t1 | | [GO:0005739](https://www.ebi.ac.uk/QuickGO/term/GO:0005739) | | C:mitochondrion | | 1 | | - | |  | |
| g607.t1 | | [GO:0005739](https://www.ebi.ac.uk/QuickGO/term/GO:0005739) | | C:mitochondrion | | 1 | | - | |  | |
| g5074.t1 | | [GO:0005739](https://www.ebi.ac.uk/QuickGO/term/GO:0005739) | | C:mitochondrion | | 1 | | - | |  | |
| g1921.t1 | | [GO:0005737](https://www.ebi.ac.uk/QuickGO/term/GO:0005737) | | C:cytoplasm | | 0.7 | | [GO:0005634 - C:nucleus (score=0.3)](https://www.ebi.ac.uk/QuickGO/term/GO:0005634) | |  | |
| g5594.t1 | | [GO:0005737](https://www.ebi.ac.uk/QuickGO/term/GO:0005737) | | C:cytoplasm | | 0.67 | | [GO:0005634 - C:nucleus (score=0.33)](https://www.ebi.ac.uk/QuickGO/term/GO:0005634) | |  | |
| g1398.t1 | | [GO:0005634](https://www.ebi.ac.uk/QuickGO/term/GO:0005634) | | C:nucleus | | 1 | | - | |  | |
| g6949.t1 | | [GO:0005737](https://www.ebi.ac.uk/QuickGO/term/GO:0005737) | | C:cytoplasm | | 1 | | - | |  | |
| g7216.t1 | | [GO:0005634](https://www.ebi.ac.uk/QuickGO/term/GO:0005634) | | C:nucleus | | 1 | | - | |  | |
| g329.t1 | | [GO:0005737](https://www.ebi.ac.uk/QuickGO/term/GO:0005737) | | C:cytoplasm | | 0.7 | | [GO:0005634 - C:nucleus (score=0.3)](https://www.ebi.ac.uk/QuickGO/term/GO:0005634) | |  | |
| g4161.t1 | | [GO:0005615](https://www.ebi.ac.uk/QuickGO/term/GO:0005615) | | C:extracellular space | | 1 | | - | |  | |
| g8262.t1 | | [GO:0005737](https://www.ebi.ac.uk/QuickGO/term/GO:0005737) | | C:cytoplasm | | 1 | | - | |  | |
| g6818.t1 | | [GO:0005737](https://www.ebi.ac.uk/QuickGO/term/GO:0005737) | | C:cytoplasm | | 0.7 | | [GO:0005634 - C:nucleus (score=0.3)](https://www.ebi.ac.uk/QuickGO/term/GO:0005634) | |  | |
| g4233.t1 | | [GO:0005739](https://www.ebi.ac.uk/QuickGO/term/GO:0005739) | | C:mitochondrion | | 1 | | - | |  | |
| g4339.t1 | | [GO:0005634](https://www.ebi.ac.uk/QuickGO/term/GO:0005634) | | C:nucleus | | 1 | | - | |  | |
| g3007.t1 | | [GO:0005739](https://www.ebi.ac.uk/QuickGO/term/GO:0005739) | | C:mitochondrion | | 0.81 | | - | |  | |
| g6530.t1 | | [GO:0005737](https://www.ebi.ac.uk/QuickGO/term/GO:0005737) | | C:cytoplasm | | 0.7 | | [GO:0005634 - C:nucleus (score=0.3)](https://www.ebi.ac.uk/QuickGO/term/GO:0005634) | |  | |
| g8300.t1 | | [GO:0005737](https://www.ebi.ac.uk/QuickGO/term/GO:0005737) | | C:cytoplasm | | 1 | | - | |  | |
| g9465.t1 | | [GO:0005737](https://www.ebi.ac.uk/QuickGO/term/GO:0005737) | | C:cytoplasm | | 0.7 | | [GO:0005634 - C:nucleus (score=0.3)](https://www.ebi.ac.uk/QuickGO/term/GO:0005634) | |  | |
| g546.t1 | | [GO:0005615](https://www.ebi.ac.uk/QuickGO/term/GO:0005615) | | C:extracellular space | | 1 | | - | |  | |
| g4668.t1 | | [GO:0005739](https://www.ebi.ac.uk/QuickGO/term/GO:0005739) | | C:mitochondrion | | 0.94 | | - | | Mitochondrial Transit Peptide | |
| g5572.t1 | | [GO:0031966](https://www.ebi.ac.uk/QuickGO/term/GO:0031966) | | C:mitochondrial membrane | | 0.79 | | - | | Mitochondrial Transit Peptide,Transmembrane Alpha Helix | |
| g1930.t1 | | [GO:0005739](https://www.ebi.ac.uk/QuickGO/term/GO:0005739) | | C:mitochondrion | | 1 | | - | |  | |
| g7752.t1 | | [GO:0005634](https://www.ebi.ac.uk/QuickGO/term/GO:0005634) | | C:nucleus | | 1 | | - | |  | |
| g6338.t1 | | [GO:0005737](https://www.ebi.ac.uk/QuickGO/term/GO:0005737) | | C:cytoplasm | | 1 | | - | |  | |
| g3939.t1 | | [GO:0005634](https://www.ebi.ac.uk/QuickGO/term/GO:0005634) | | C:nucleus | | 1 | | - | |  | |
| g224.t1 | | [GO:0005737](https://www.ebi.ac.uk/QuickGO/term/GO:0005737) | | C:cytoplasm | | 1 | | - | |  | |
| g3066.t1 | | [GO:0005737](https://www.ebi.ac.uk/QuickGO/term/GO:0005737) | | C:cytoplasm | | 0.7 | | [GO:0005634 - C:nucleus (score=0.3)](https://www.ebi.ac.uk/QuickGO/term/GO:0005634) | |  | |
| g392.t1 | | [GO:0005615](https://www.ebi.ac.uk/QuickGO/term/GO:0005615) | | C:extracellular space | | 0.96 | | - | | Signal Peptide | |
| g7271.t1 | | [GO:0012505](https://www.ebi.ac.uk/QuickGO/term/GO:0012505) | | C:endomembrane system | | 0.87 | | [GO:0031090 - C:organelle membrane (score=0.42)](https://www.ebi.ac.uk/QuickGO/term/GO:0031090) | | Transmembrane Alpha Helix | |
| g1111.t1 | | [GO:0005739](https://www.ebi.ac.uk/QuickGO/term/GO:0005739) | | C:mitochondrion | | 1 | | - | |  | |
| g5622.t1 | | [GO:0005737](https://www.ebi.ac.uk/QuickGO/term/GO:0005737) | | C:cytoplasm | | 0.7 | | [GO:0005634 - C:nucleus (score=0.3)](https://www.ebi.ac.uk/QuickGO/term/GO:0005634) | |  | |
| g7744.t1 | | [GO:0005886](https://www.ebi.ac.uk/QuickGO/term/GO:0005886) | | C:plasma membrane | | 0.82 | | [GO:0012505 - C:endomembrane system (score=0.29)](https://www.ebi.ac.uk/QuickGO/term/GO:0012505) | | Transmembrane Alpha Helix | |
| g3937.t1 | | [GO:0005737](https://www.ebi.ac.uk/QuickGO/term/GO:0005737) | | C:cytoplasm | | 1 | | - | |  | |
| g3117.t1 | | [GO:0005739](https://www.ebi.ac.uk/QuickGO/term/GO:0005739) | | C:mitochondrion | | 0.94 | | - | | Mitochondrial Transit Peptide | |
| g2430.t1 | | [GO:0005739](https://www.ebi.ac.uk/QuickGO/term/GO:0005739) | | C:mitochondrion | | 0.56 | | - | | Mitochondrial Transit Peptide | |
| g8410.t1 | | [GO:0005737](https://www.ebi.ac.uk/QuickGO/term/GO:0005737) | | C:cytoplasm | | 1 | | - | |  | |
| g5740.t1 | | [GO:0005615](https://www.ebi.ac.uk/QuickGO/term/GO:0005615) | | C:extracellular space | | 1 | | - | |  | |
| g2854.t1 | | [GO:0005737](https://www.ebi.ac.uk/QuickGO/term/GO:0005737) | | C:cytoplasm | | 1 | | - | |  | |
| g3571.t1 | | [GO:0005615](https://www.ebi.ac.uk/QuickGO/term/GO:0005615) | | C:extracellular space | | 0.86 | | - | |  | |
| g6117.t1 | | [GO:0005737](https://www.ebi.ac.uk/QuickGO/term/GO:0005737) | | C:cytoplasm | | 1 | | - | |  | |
| g3978.t1 | | [GO:0005737](https://www.ebi.ac.uk/QuickGO/term/GO:0005737) | | C:cytoplasm | | 1 | | - | |  | |
| g2178.t1 | | [GO:0005737](https://www.ebi.ac.uk/QuickGO/term/GO:0005737) | | C:cytoplasm | | 1 | | - | |  | |
| g3897.t1 | | [GO:0005615](https://www.ebi.ac.uk/QuickGO/term/GO:0005615) | | C:extracellular space | | 1 | | - | |  | |
| g7841.t1 | | [GO:0005737](https://www.ebi.ac.uk/QuickGO/term/GO:0005737) | | C:cytoplasm | | 0.7 | | [GO:0005634 - C:nucleus (score=0.3)](https://www.ebi.ac.uk/QuickGO/term/GO:0005634) | |  | |
| g5567.t1 | | [GO:0005737](https://www.ebi.ac.uk/QuickGO/term/GO:0005737) | | C:cytoplasm | | 0.7 | | [GO:0005634 - C:nucleus (score=0.3)](https://www.ebi.ac.uk/QuickGO/term/GO:0005634) | |  | |
| g376.t1 | | [GO:0012505](https://www.ebi.ac.uk/QuickGO/term/GO:0012505) | | C:endomembrane system | | 0.89 | | [GO:0005886 - C:plasma membrane (score=0.35)](https://www.ebi.ac.uk/QuickGO/term/GO:0005886) | |  | |
| g831.t1 | | [GO:0005737](https://www.ebi.ac.uk/QuickGO/term/GO:0005737) | | C:cytoplasm | | 1 | | - | |  | |
| g1298.t1 | | [GO:0005886](https://www.ebi.ac.uk/QuickGO/term/GO:0005886) | | C:plasma membrane | | 0.55 | | [GO:0012505 - C:endomembrane system (score=0.46)](https://www.ebi.ac.uk/QuickGO/term/GO:0012505) | |  | |
| g5075.t1 | | [GO:0005615](https://www.ebi.ac.uk/QuickGO/term/GO:0005615) | | C:extracellular space | | 1 | | - | |  | |
| g689.t1 | | [GO:0005615](https://www.ebi.ac.uk/QuickGO/term/GO:0005615) | | C:extracellular space | | 0.93 | | - | |  | |
| g5703.t1 | | [GO:0005634](https://www.ebi.ac.uk/QuickGO/term/GO:0005634) | | C:nucleus | | 1 | | - | |  | |
| g246.t1 | | [GO:0005615](https://www.ebi.ac.uk/QuickGO/term/GO:0005615) | | C:extracellular space | | 0.87 | | - | |  | |
| g7536.t1 | | [GO:0005615](https://www.ebi.ac.uk/QuickGO/term/GO:0005615) | | C:extracellular space | | 1 | | - | |  | |
| g1243.t1 | | [GO:0005615](https://www.ebi.ac.uk/QuickGO/term/GO:0005615) | | C:extracellular space | | 1 | | - | |  | |
| g336.t1 | | [GO:0005737](https://www.ebi.ac.uk/QuickGO/term/GO:0005737) | | C:cytoplasm | | 0.7 | | [GO:0005634 - C:nucleus (score=0.3)](https://www.ebi.ac.uk/QuickGO/term/GO:0005634) | |  | |
| g1848.t1 | | [GO:0005634](https://www.ebi.ac.uk/QuickGO/term/GO:0005634) | | C:nucleus | | 1 | | - | |  | |
| g5531.t1 | | [GO:0005615](https://www.ebi.ac.uk/QuickGO/term/GO:0005615) | | C:extracellular space | | 1 | | - | |  | |
| g6280.t1 | | [GO:0005737](https://www.ebi.ac.uk/QuickGO/term/GO:0005737) | | C:cytoplasm | | 1 | | - | |  | |
| g4038.t1 | | [GO:0005739](https://www.ebi.ac.uk/QuickGO/term/GO:0005739) | | C:mitochondrion | | 1 | | - | |  | |
| g4533.t1 | | [GO:0005737](https://www.ebi.ac.uk/QuickGO/term/GO:0005737) | | C:cytoplasm | | 1 | | - | |  | |
| g4131.t1 | | [GO:0005737](https://www.ebi.ac.uk/QuickGO/term/GO:0005737) | | C:cytoplasm | | 1 | | - | |  | |
| g2871.t1 | | [GO:0012505](https://www.ebi.ac.uk/QuickGO/term/GO:0012505) | | C:endomembrane system | | 0.68 | | [GO:0005886 - C:plasma membrane (score=0.54)](https://www.ebi.ac.uk/QuickGO/term/GO:0005886) | |  | |
| g8909.t1 | | [GO:0005615](https://www.ebi.ac.uk/QuickGO/term/GO:0005615) | | C:extracellular space | | 1 | | - | |  | |
| g7137.t1 | | [GO:0005737](https://www.ebi.ac.uk/QuickGO/term/GO:0005737) | | C:cytoplasm | | 1 | | - | |  | |
| g7702.t1 | | [GO:0005737](https://www.ebi.ac.uk/QuickGO/term/GO:0005737) | | C:cytoplasm | | 1 | | - | |  | |
| g2793.t1 | | [GO:0005739](https://www.ebi.ac.uk/QuickGO/term/GO:0005739) | | C:mitochondrion | | 1 | | - | |  | |
| g1419.t1 | | [GO:0005737](https://www.ebi.ac.uk/QuickGO/term/GO:0005737) | | C:cytoplasm | | 0.7 | | [GO:0005634 - C:nucleus (score=0.3)](https://www.ebi.ac.uk/QuickGO/term/GO:0005634) | |  | |
| g6131.t1 | | [GO:0005737](https://www.ebi.ac.uk/QuickGO/term/GO:0005737) | | C:cytoplasm | | 1 | | - | |  | |
| g6981.t1 | | [GO:0005737](https://www.ebi.ac.uk/QuickGO/term/GO:0005737) | | C:cytoplasm | | 0.81 | | [GO:0005634 - C:nucleus (score=0.19)](https://www.ebi.ac.uk/QuickGO/term/GO:0005634) | |  | |
| g7166.t1 | | [GO:0005739](https://www.ebi.ac.uk/QuickGO/term/GO:0005739) | | C:mitochondrion | | 1 | | - | |  | |
| g4791.t1 | | [GO:0005615](https://www.ebi.ac.uk/QuickGO/term/GO:0005615) | | C:extracellular space | | 1 | | - | |  | |
| g3046.t1 | | [GO:0005737](https://www.ebi.ac.uk/QuickGO/term/GO:0005737) | | C:cytoplasm | | 1 | | - | |  | |
| g4154.t1 | | [GO:0005615](https://www.ebi.ac.uk/QuickGO/term/GO:0005615) | | C:extracellular space | | 1 | | - | |  | |
| g2250.t1 | | [GO:0005737](https://www.ebi.ac.uk/QuickGO/term/GO:0005737) | | C:cytoplasm | | 1 | | - | |  | |
| g1009.t1 | | [GO:0005615](https://www.ebi.ac.uk/QuickGO/term/GO:0005615) | | C:extracellular space | | 1 | | - | |  | |
| g1387.t1 | | [GO:0005737](https://www.ebi.ac.uk/QuickGO/term/GO:0005737) | | C:cytoplasm | | 1 | | - | |  | |
| g6048.t1 | | [GO:0005634](https://www.ebi.ac.uk/QuickGO/term/GO:0005634) | | C:nucleus | | 1 | | - | |  | |
| g8255.t1 | | [GO:0005737](https://www.ebi.ac.uk/QuickGO/term/GO:0005737) | | C:cytoplasm | | 1 | | - | |  | |
| g8671.t1 | | [GO:0005737](https://www.ebi.ac.uk/QuickGO/term/GO:0005737) | | C:cytoplasm | | 1 | | - | |  | |
| g7238.t1 | | [GO:0005615](https://www.ebi.ac.uk/QuickGO/term/GO:0005615) | | C:extracellular space | | 1 | | - | |  | |
| g4993.t1 | | [GO:0005634](https://www.ebi.ac.uk/QuickGO/term/GO:0005634) | | C:nucleus | | 1 | | - | |  | |
| g4525.t1 | | [GO:0005615](https://www.ebi.ac.uk/QuickGO/term/GO:0005615) | | C:extracellular space | | 1 | | - | |  | |
| g4740.t1 | | [GO:0005737](https://www.ebi.ac.uk/QuickGO/term/GO:0005737) | | C:cytoplasm | | 1 | | - | |  | |
| g827.t1 | | [GO:0005737](https://www.ebi.ac.uk/QuickGO/term/GO:0005737) | | C:cytoplasm | | 1 | | - | |  | |
| g2865.t1 | | [GO:0005739](https://www.ebi.ac.uk/QuickGO/term/GO:0005739) | | C:mitochondrion | | 1 | | - | |  | |
| g4750.t1 | | [GO:0005615](https://www.ebi.ac.uk/QuickGO/term/GO:0005615) | | C:extracellular space | | 1 | | - | |  | |
| g3654.t1 | | [GO:0005739](https://www.ebi.ac.uk/QuickGO/term/GO:0005739) | | C:mitochondrion | | 1 | | - | |  | |
| g5764.t1 | | [GO:0005737](https://www.ebi.ac.uk/QuickGO/term/GO:0005737) | | C:cytoplasm | | 0.7 | | [GO:0005634 - C:nucleus (score=0.3)](https://www.ebi.ac.uk/QuickGO/term/GO:0005634) | |  | |
| g2300.t1 | | [GO:0012505](https://www.ebi.ac.uk/QuickGO/term/GO:0012505) | | C:endomembrane system | | 0.85 | | [GO:0005886 - C:plasma membrane (score=0.31)](https://www.ebi.ac.uk/QuickGO/term/GO:0005886) | | Transmembrane Alpha Helix | |
| g3069.t1 | | [GO:0005737](https://www.ebi.ac.uk/QuickGO/term/GO:0005737) | | C:cytoplasm | | 0.7 | | [GO:0005634 - C:nucleus (score=0.3)](https://www.ebi.ac.uk/QuickGO/term/GO:0005634) | |  | |
| g7438.t1 | | [GO:0005634](https://www.ebi.ac.uk/QuickGO/term/GO:0005634) | | C:nucleus | | 1 | | - | |  | |
| g9173.t1 | | [GO:0005615](https://www.ebi.ac.uk/QuickGO/term/GO:0005615) | | C:extracellular space | | 0.98 | | - | | Signal Peptide | |
| g567.t1 | | [GO:0005737](https://www.ebi.ac.uk/QuickGO/term/GO:0005737) | | C:cytoplasm | | 0.7 | | [GO:0005634 - C:nucleus (score=0.3)](https://www.ebi.ac.uk/QuickGO/term/GO:0005634) | |  | |
| g2952.t1 | | [GO:0005739](https://www.ebi.ac.uk/QuickGO/term/GO:0005739) | | C:mitochondrion | | 0.78 | | - | | Mitochondrial Transit Peptide | |
| g726.t1 | | [GO:0005739](https://www.ebi.ac.uk/QuickGO/term/GO:0005739) | | C:mitochondrion | | 0.65 | | - | | Mitochondrial Transit Peptide | |
| g1996.t1 | | [GO:0005737](https://www.ebi.ac.uk/QuickGO/term/GO:0005737) | | C:cytoplasm | | 1 | | - | |  | |
| g6621.t1 | | [GO:0005737](https://www.ebi.ac.uk/QuickGO/term/GO:0005737) | | C:cytoplasm | | 1 | | - | |  | |
| g7535.t1 | | [GO:0005739](https://www.ebi.ac.uk/QuickGO/term/GO:0005739) | | C:mitochondrion | | 0.9 | | - | | Mitochondrial Transit Peptide | |
| g8183.t1 | | [GO:0005615](https://www.ebi.ac.uk/QuickGO/term/GO:0005615) | | C:extracellular space | | 1 | | - | |  | |
| g6990.t1 | | [GO:0005737](https://www.ebi.ac.uk/QuickGO/term/GO:0005737) | | C:cytoplasm | | 0.7 | | [GO:0005634 - C:nucleus (score=0.3)](https://www.ebi.ac.uk/QuickGO/term/GO:0005634) | |  | |

^a^ Protein accession number in the *K. chersonesos* database of ab initio translated proteins

^b^ Alternative localization (if any): it corresponds to the second most-probable compartment with a prediction score greater than 0.1.

**Supplementary Figure S5**

**
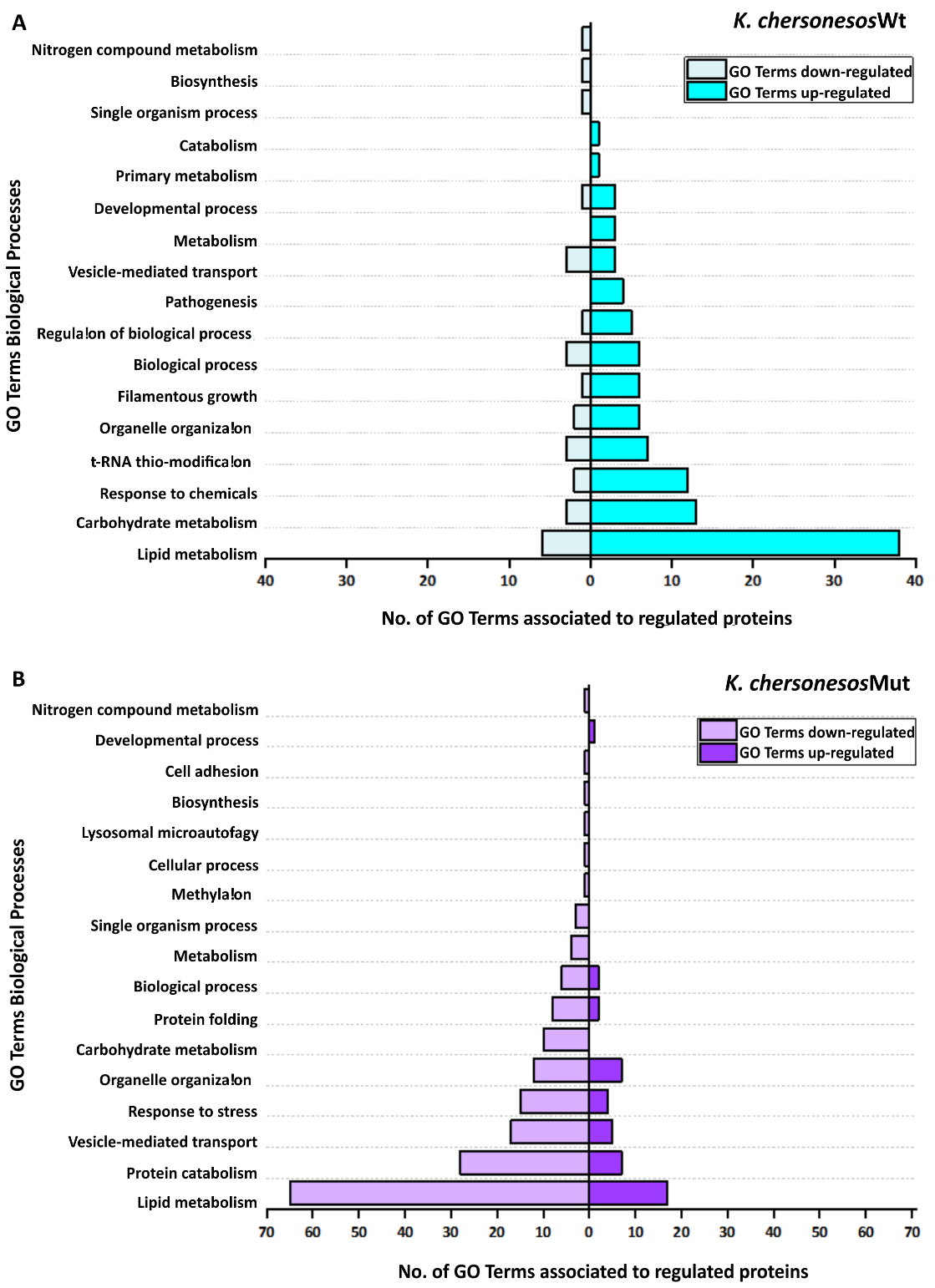
**

**Supplementary Figure S5_** Biological processes GO terms of differentially expressed proteins in *K. chersonesos* at minimal medium condition, after exposure to PBAT. (a) *K. chersonesos* wild type, (b) *K. chersonesos* mutant. Proteins with changed abundance (fold change ≥ 2, p ≤ 0.05) were annotated with terms representing biological processes using BLAST2GO Pro and subsequently summarized using REVIGO. All graphs were created using Origin Pro v 9.5 (<https://www.originlab.com/origin>).

**Supplementary Table S4**

**Supplementary Table 4_** Relative abundance of proteins involved in carbohydrate and lipid metabolism and in stress response in *K. chersonesos* Wt and Mut upon exposure to PBAT.

| ***Knufia chersonesos* Wt, minimal medium** | | | | | | |
| --- | --- | --- | --- | --- | --- | --- |
| ***Protein* accession No.^a^** | **UniProtKb accession No.** | **UniProtKb Protein name** | **Max Score** | **Identity (%)** | **Expected value** | **FC^b^** |
| g7863.t1 | A0A1C1D0J4_9EURO | Glycoside hydrolase family 16 protein *(Cladiophialophora carrionii)* | 601 | 49.4 | 1.1E-72 | 100 |
| g7511.t1 | C9SYD2_VERA1 | Xyloglucan-specific endoglucanase (**Verticillium alfalfae VaMs.102**) | 977 | 58.7 | 1.1E-129 | 100 |
| g4716.t1 | A0A179G0Y3_PURLI | Muramidase *(Purpureocilium lilacinum)* | 536 | 51.8 | 9.9E-63 | 22.23 |
| g4930.t1 | W9Y456_9EURO | Murein transglycosylase *(Capronia epimyces CBS606.96)* | 1,889 | 54.5 | 0 | 5.84 |
| g4842 | H6BU59_EXODN | Cathepsin D (*Exophiala dermatitidis* CBS 525.76) | 1,211 | 58 | 1.8E-156 | 4.91 |
| g8540.t1 | A0A0D2FAU6_9EURO | **1,3-beta-glucanosyltransferase** *([Phialophora americana](https://www.uniprot.org/taxonomy/5601))* | 1,578 | 61.6 | 0 | 4.07 |
| g488.t1 | A0A179FFJ3_METCM | **Repressible acid phosphatase (Pochonia chlamydosporia 170)** | 1,632 | 64.2 | 0 | 3.31 |
| g7971.t1 | W9XFN3_9EURO | Endo-1,3(4)-beta-glucanase *(Cladophialophora psammophila CBS110553)* | 1,250 | 50.7 | 1.5E-165 | 3.19 |
| g1975.t1 | W9WT35_9EURO | Glucan 1,3-beta-glucosidase *(Cladophialophora psammophila CBS110553)* | 1,325 | 76.6 | 0 | 2.98 |
| g6247.t1 | A0A0D2AQ38_9EURO | Lysophospholipase *(Exophiala oligosperma)* | 2,090 | 61 | 0 | 2.95 |
| g7566.t1 | A0A1Y2EA24_9PEZI | Carboxylic ester hydrolase (*Pseudomassariella vexata*) | 1,837 | 58.1 | 0 | 2.93 |
| g7569.t1 | A0A0D2AG04_9PEZI | Cutinase (*Verruconis gallopava*) | 704 | 54.0 | 3.3E-90 | 2.78 |
| g6203.t1 | H6BNZ6_EXODN | Extracellular cell wall glucanase Crf1 *(Exophiala dermatitidis CBS525.76)* | 1,216 | 57.2 | 4.7E-163 | 2.66 |
| g3601.t1 | A0A1C1D0Ja_9EURO | Glycoside hydrolase family 16 protein (*Cladiophialophora carrionii*) | 609 | 46.8 | 7.9E-74 | 2.62 |
| g5038 | (A0A0D2C082_9EURO) | 1,3-beta-glucanosyltransferase *(Exophiala xenobiotica)* | 1,855 | 79.8 | 0 | 2.60 |
| g583.t1 | (A0A0D2F228_9EURO) | 1,3-beta-glucanosyltransferase *(Exophiala xenobiotica)* | 1,995 | 68.6 | 0 | 2.55 |
| g8924.t1 | A0A0D2KI27_9EURO | Mannan endo-1,6-alpha-mannosidase *(Fonsecaea multimorphosa CBS 102226)* | 1,864 | 72.7 | 0 | 2.54 |
| g8444.t1 | H6BWF7_EXODN | **Acid phosphatase** (*Exophiala dermatitidis* CBS 525.76) | 1,619 | 68.2 | 0 | 2.49 |
| g7970.t1 | H6C6J4_EXODN | Endoglucanase *(Exophiala dermatitidis CBS525.76)* | 925 | 47.8 | 8.7E-118 | 2.42 |
| g2279.t1 | A0A1J9RJA8_9PEZI | Carboxylic ester hydrolase (*Diplodia corticola*) | 1,819 | 63.0 | 0 | 2.38 |
| g4729.t1 | A0A0D2C3R4_9EURO | Glycosidase *(Exophiala xenobiotica)* | 1,264 | 55.4 | 1E-165 | 2.32 |
| g4369.t1 | A0A1J7IST7_9PEZI | Amidase signature enzyme *(Coniochaeta ligniaria NRRL 30616)* | 2,016 | 73.1 | 0 | 2.15 |
| g3607.t1 | A0A0D2CPX4_9EURO | Glycerophosphoryl diester phosphodiesterase *(Capronia coronata CBS617.96)* | 1,587 | 68.9 | 0 | 2.13 |
| g1109.t1 | A0A2K3Q6V2_9HYPO | Secretory lipase (*Tolypocladium capitatum*) | 1,134 | 48.8 | 3E-148 | 2.10 |
| g3201.t1 | A0A074VZN4_9PEZI | 3-carboxy-cis,cis-mucoante lactonizing enzyme *(Aerobasidium melanogenum CBS110374)* | 1,161 | 57.1 | 9.7E-155 | 2.10 |
| g689.t1 | H6C6J4_EXODN | Endoglucanase *(Exophiala dermatitidis CBS525.76)* | 911 | 59.9 | 4.4E-118 | ̶ 100 |
| g5495.t1 | A0A364MWV0_9PLEO | Arabinan endo-1,5-alpha-L-arabinosidase *(Stemphylium lycopersici)* | 1,166 | 70.4 | 5.1E-158 | **̶ 7.75** |
| g1827.t1 | A0A0S7DK79_9EURO | Non-reducing end alpha-L-arabinofuranosidase BoGH43B *(Aspergillus lentulus)* | 1,735 | 62.0 | 0 | **̶ 8.33** |
| ***Knufia chersonesos* Mut, minimal medium** | | | | | | |
| g4295.t1 | W2RQJ3_9EURO | Cutinase *(Cyphellophora europaea CBS 101466)* | 506 | 48.4 | 4.2E-61 | 100 |
| g4619.t1 | A0A1Y2VIA8_9PEZI | Glycoside hydrolase family 28 protein *(Hypoxylon sp. CO27-5)* | 1,352 | 69.1 | 0 | 100 |
| g8915.t1 | A0A1L7WTP0_9HELO | Carboxylic ester hydrolase *(Phialocephala subalpine)* | 1,256 | 44.5 | 1.3E-164 | 100 |
| g4772.t1 | A0A0N1HLI9_9EURO | Protein SSD1 *(Phialophora attae)* | 5,115 | 74.8 | 0 | 100 |
| g4392.t1 | W9Z6G9_9EURO | Fibrillarin-like pre-rRNA processing protein *(Exophiala dermatitidis CBS525.76)* | 1,394 | 84.6 | 0 | 100 |
| g5720.t1 | V9DRM7_9EURO | Eukaryotic translation initiation factor 3 subunit I *(Cladiophialophora carrionii)* | 1,545 | 83.9 | 0 | 100 |
| g8970.t1 | A0A0D2BRZ4_9EURO | Peptidyl-prolyl cis-trans isomerase *(Exophiala xenobiotica)* | 871 | 89 | 6.6E-117 | 100 |
| g395.t1 | W9XT49_9EURO | Cysteinyl-tRNA synthetase *(Capronia coronate CBS617.96)* | 2,767 | 65.1 | 0 | 100 |
| g4716.t1 | A0A179G0Y3_PURLI | Muramidase *(Purpureocillium lilacinum)* | 536 | 51.8 | 9.98E-63 | 5.33 |
| g7863.t1 | A0A1C1D0J4_9EURO | Glycoside hydrolase family 16 protein *(Cladiophialophora carrionii)* | 601 | 49.4 | 1.1E-72 | 37.53 |
| g5667.t1 | A0A0D2BK02_9EURO | Ribosomal protein *(Exophiala xenobiotica)* | 1,043 | 93.5 | 8.4E-143 | **̶ 10.52** |
| g6685.t1 | A0A0N1HE03_9EURO | Endochitinase B1 *(Phialophora attae)* | 1,412 | 62.8 | 0 | **̶ 20.40** |
| g7588.t1 | H6CB67_EXODN | H/ACA ribonucleoprotein complex subunit 2 *(Exophiala dermatitidis CBS525.76)* | 750 | 58.2 | 1.7E-96 | ̶ ,100 |
| g3571.t1 | A0A0D2AN11_9PEZI | 60S acidic ribosomal protein P2 *(Verruconis gallopava)* | 496 | 89.4 | 8.6E-63 | ̶ 100 |
| g1848.t1 | W9WJM3_9EURO | Large subunit ribosomal protein L35e *(Cladophialophora yegresii CBS114405)* | 546 | 85.7 | 5.9E-70 | ̶ 100 |
| g5594.t1 | H6C8P0_EXODN | Triacylglycerol lipase *(Exophiala dermatitidis CBS525.76)* | 542 | 39.7 | 3.8E-63 | ̶ 100 |
| g4233.t1 | A0A438MZU1_EXOME | L-xylo-3-hexulose reductase*(Hypocrea jecorina)* | 1,262 | 74.9 | 4:5e-173 | ̶ 100 |
| g376.t1 | W9YAZ0_9EURO | Beta-glucosidase *(Capronia epimyces CBS606.96)* | 3,332 | 66.4 | 0 | ̶ 100 |
| g689.t1 | H6C6J4_EXODN | Endoglucanase *(Exophiala dermatitidis CBS525.76)* | 911 | 59.9 | 4.4E-118 | ̶ 100 |
| g9173.t1 | A0A0G2FDQ5_9PEZI | Putative glycoside hydrolase family 61 protein *(Diaporthe ampelina)* | 921 | 49.9 | 6.7E-119 | ̶ 100 |
| g2178.t1 | A0A0D1X661_9EURO | TIGR01456 family HAD hydrolase *(Exophiala sideris)* | 1,797 | 75 | 0 | ̶ 100 |

^a^ Protein accession number in the *K. chersonesos* database of *ab initio* translated proteins

^b^Up- and down-regulation of proteins (expressed as fold change) detected in the PBAT-exposed secretomes of *K. chersonesos* Wt and Mut grown in minimal media compared to the unexposed secretomes. Fold change equal to 100 or -100 indicates proteins exclusively found in one out of the two experimental conditions (i.e. On/Off proteins).

**Sequences of the polyesterases of interest** (in order of appearance in Table 3)

**>g6247.t1**

MKFTICAAVLGSVASIAQATVVSPDVPRNAPNALSNLRRALPASPKGYTPSEVDCPSTAPSVRLADSLSPNETEWVQRRRDNTVEPMRDFLSRMNISNFDAGQYIDDHRNNVSALPNIGIAFSGGGWRALINGAGVLSAFDSRSDNSTNTGQLGGLLQSATYIAGLSGGNWLVGSIYINNFTTVSALRDDPSVWQFDNSILEGPPSGGIQFLNTASYYSDLYDEVEGKINAGFDASLTDYWGRALSYQLINATDGGPAYTWSSIAQTEGFQNAEQPFPISLADARAPGQKIVSLNSSNFEFNPFEMGTWDPTTYGFIPTRYLGTNMTNGSVADDSRCTIGFDNAGFVMGTSSSLFNTLILQLNDSSLDIPDILRDAVGSLLVNLGSNDNDIADYDPNPFYGWNPTGNARSATNRSLTLVDGGEDNQNIPFNPLIQPLREVDVIFASDNSADTPTNWPNGSALVQTYMRSLEPISNGTAFPSIPDVNTFINLGLNYRPTFFGCNASNITRSSPSLDVSDRAVTPPLIVYIPNSPYVTYSNQSTVTLETNNTYRDAMIRNGYEVATMANGTLSGHENWSQCVACAILSRSFDRTGTEVPQACQDCFSQYCWDGTLNSTNPGTYTPEPRLSTLDIDSAGTLSAHFSPGVMAVAALGSALALIL

**>g1109.t1**

MLLSILLPLMFCIACQAQQQRLMPQSDIWNSTYEVTQDRARGANLTDAELHDISVALNFERSNWATGSVADDEFYTLPSNASSASPGSVVKVQAYTNTSTYTLPPNTALSRIIFMTEDLNGTAVPASAYVLWPYLPRTQADGRYPLVTWGHGTSGGFAECGPSHIRNLWYQYSAPYALALAGYVVVAPDYRGLGINETANGKPIYHSYGAGQSAGIDLLYAAQAAQSAFPSISENFVVMGHSQGGNAAWGAAVRQAQSPSAGYLGTIAGSPTTNYTAIIEFYSGNPIIPPQLQLLWANALRGLHPSFNLSTILTNTGIARLNLASELGMCNSAVGMLLPSGSSTYVVQPDWLSVPELSSYLSLLDRGTQQEVAGPMLVLQGTLDPAVPEQITTAAVQSTCKLYPTSDIEYWLFANVTHVPVLYASQRLWLDWIAERFAAADNAEDSNGAGSDGSCSMKNFTSGPMPSENYQTELEYYLELATQGHQVA

**>g2279.t1**

MSTLRNLVRFSLLGLPLYQSVAAVSDPVVEVEHVGSYRGTVSEYVDGVNVFKGIRYADPPTGQYRWAHPPHPASFSGVKNATTYGNSCPSGGIGPGSSSASEDCLFLNVWTPEGFTNTSNYPVFFWIYGGRFESGAGSDLTYDGSGLAKQGVVVVTMNYRLGALGFLAHPELSEETGYNGSGNWGLMDQQAALHWTNENIQNFGGNSSQITLGGQSAGAASVLDQVYSPQASGLFQGAIAESGARAPHDPLTGSLASSHLKLSAAEAHGVTFFNQLNVSTVAQARNLSLSTILGARTPSSTLFVGTPFADNEAYIEPPYFRPNIDNYILTLSYADSLALNNHSDVPILTGNNKDESGAAPNPGVNITTYKSNNEAIFEPLGLADEFFNIWPASNDNEANNQTNNFYRNQSLVSTWLWADAWAAGGAKNNVYTYYWTHAPPGQTSGVFHGSEINYALDNMPYGTTLTGQTLNWTSTDYKIADVMSSYWINFISTGNPNGDSLTHWAPSTNATKTTMMLGDGWGTIPIASEKVIEFIEDYFSQEPAY

**>g7566.t1**

MASSGLLWALLATSALAQSLADVCTVDYVVDHLPSDDDFNGLVFDSSSVTANPVYNASANGQNNFVSATGLNYCNVTFSYSHAGRSNDTFNLWYYLPTPSSFQNRFLATGGGGESINSGASGLAQGIINGAVAGLTDGGFGGFNNEVTDVILLANGTLNYEALYSFGYKGIHEMTIVGKQLAKNFYNTSSIYSYYFGCSEGGREGWSQVQRYATQFDGAVIGAPAIRQAYQQVNHLSSGVIETQMNYFPPPCELERILNDTIAFCDPLDGKTDGVVSRTDLCRLQFNANMSIGNSYYCAASGGMGGGMGGGPTKRQFGGTTPAQNGTVTAEGAAVAQSIVNGLFDSEGRQIYMSYQPSATFSDAQTTYNDTSDEWYVTADGIAVTWINMFLDEVSSSELSLNNVTADTLRGWILEGLQKYSGTMQTVWPDLEEFHEAGGKVLTYHGESDYSIPTISSVIYQNAVRQTLYPNMSYNEGMDKINEWYRLFLVPGAGHCGPSDTQPNGPWPLTTLETLIQWVEDDVKPVTLNGTVTEGSEEGDSQQICAFPLRPHWAGNDSSVTCIYPDEAALDTWYPDLNSIPLPVY

**>g7569.t1**

MLSTSLSGLAALYLATISSASPFPANNIAQRQDTTACEAVHIFLARGWNVSDYPGRQGALAGSICYGLDSCGYEDIYYYNLDDVPYCQGVTDGVANGTAAIKDYAARCPDSQIVLSGYSQGANVVGNILGGGGGEFSNCTIAETSGLDVNSAAGKQIAVVTLFGDPLHVGGEYYNVLGGAPYNSSDPRDATSLAKLNQYAPVLRSYCQESDPICAGGGPGPFNISDHLNYFELYTQVAAGWIKWVLENPQK

**>g4295.t1**

MLLTTILAALFATALSVPLPGGQSPTPDEIRQAATTRNELLNGPCRNVTVIFARGTTESGNIGSLVGPALESALDARLGSTTVAFQGVDYEANVAGYYAGGSDTGASTMASLVNVASSKCPSTQIVLSGYSQGAQVVYKAAAQLTPALTAQVKAAVLFGNPYNGLPVPNIDNSTTWTFCHAGDLICQGQAVVTPAHLDYAADTPAAAAYIASRVTLNMS

**>g8915.t1**

MLSFVLLLAASCAAQSVTLPGGTLQGGQCATSNASYFYSVPYAQPPTGDLRFAAPQPYNGTLGQATAPSPRCVQFGQAFIEYPYSEDCLYLDVWVPPNANNLPVKVWIYGGGNSAGSISNPTYNGCNLATDSIVVSVNYRLGPLGFLGLQDFGIQGNMGIQDQLLGLSWVQDNIQAFGGDPSKVVLFGESAGAVDTYVIGTLPQAPSLLQAGIAQSGGGRGLPSSGPANDFGARWANSLNCSDAACLRSLSVEQLNVTLPTPTGPTINPASVSGFGPFVDGEIVPADPAEVGVQVPFIFGSNTMEGTLLILSRSGVTGPQDITEAVYLAYLNSTYGSGASTVAEQYPVSAFNSTPFPAFYALVQVYTATSFWCPALAGLERAAQKGVPVWTYRWGQAPSCPWYNVFPPGVLPIFGAAHTAEIPFVFANVDNNPPPDGTCSFTAAEKSLSRQMVGFWTSMAANGYPGSVWPQFQTDGTPGINVINGSSSVTPGMVDYSACSFLQQAGAAALGNSSAGATSSSAGGPIATYVSGAESTLAGFSYVVAIVAIMGACTLLM

**>g1329.t1**

MRTTSQLLLAGALSGVCLAAPAAQNFARQEGSGSSPTASIDSGVVVGIQTSVANSPNLVNQYLGVPFAASPTRFAPAQTATPWSEPYQATQRGPACIQQFNYPELSRNFTIAAFNTPAPQESEDCLTVNIFTPANAQTGGALKPVMFWIYGGALQFGYNGNAAYDGSSFAANEDVIVVATNYRTNVFGFPSSPELPLTERNLGYLDQRFALAWVQRNIASFGGDPSRVTIFGESAGSFSVDSLVTSFGPSAPDGPPPFHAAIMESGQSSVSARYPADPASWDSLLAALNCTTPSDGLACARAAPAATLKSVIEHLALSFRPTNDNYTQLTYPEAARAAGDIARVPVITGTNANEGILFTFTNNNTEAFLRTTFNNLLTDAQIQAIIAAYPIGARGIASSTEQIAAIYTELGFQCPAAIVANDSAAAGIPTWRYYYNASFAQLQPIPGINFGAWHSSEIPLVFGTYASTGADIGARGASLSALMQNMWATFAKNPMGGPVGSWDEVGDAGFVEVIGGPGGLNSQGMLRRAGSAAEVDGGRCEIWRPVYRL

**>g2917.t1**

MSQTSATGKAILAGTLLLTLAFASKPSPSHTTDTSGCGRVHAPGYHDADDTNSLESGGLTRQYGIYVPQSYNDNPNKPRKLILDYHGNNGTPLNQYNNSRYFDYPNGEEYLAVYPAGVDQSFQSAPYATEGVDDLQFTTDLLAHLRQNYCVDSDHVYASGKSNGGGFVDFLACSENGDEFAAFAMASAALYSDNGVEQCNNTRPRAVLESHGVNDTTISYYGGPRNGATLPDIQTWIGWWAQRDGCKAGCEDCRDVQQHSGYEVISYDCGGLRDVVQHYEVFDLGHCWPSSTGDNSDSARAYCGDRSLDFTPVVLDFFSRWSLRSISAKWREWET

**>g4612.t1**

MPYTITTLLALAASLSATNAALYNTVIETKNGPVQGYPAFNSTPTGGLTHWKDITVWKGIPFAASTGGENRFRPPQPVTSWNTTLDAKNFGAVCPSATGGMGNSYTIDEDCLNLNVWSAANSTDAKLPVVMWSYPAESTAADDLFNGGGMADKGVVYVNYNYRTGSFGWLALPELSEERLAEKGVNSSGNWGMLDQFAALEWIKTNIAAFGGDPEHVTVMGQSAGSAATYHIVNSPLTKGQIVGAIIESGVRDPHDPLAGSLAENYLTLESALELGVNYTKSLNATTLAQLRALPMEDLVTQFMSTWDFTATLDYYAMPATYLETLLHGAANNVPILTGNTKDESGATYGLNISLSTYLSELNSTYNGTWAEDFFALYPANNSATASAAYNMQFTDRSKVGTWLWAQLWYTASNNPVYTYLWDHAPPGQSQGAYHESEINYVLNNLYDTDKPWTSEDYEIAAKMNEYWVNFIKTGNPNGGNLTTWVPSSENPVTKQLGNGWGPIPVASAEQVLLFEAWFDSLPAY

**>g7247.t1**

MHWLAPFTAGLVPLLSLTSAVSVQSRQTTACNNSPDLCSKSYSEITYLGAHDSPFVRDASTGYSTSGNQYYNSTVQLSAGVRLLSAQVHESNGDWHLCHSSCDLLDAGLLSDWLEDIKDWIDANPNQVVTLLLVNSDSATADQLGAEFTTAGITSLAYKPASNTSPPTSWPTLQELIDADTPLLVFVASLDTASTTTETAYLMDEFTFIFENSYENTSPSHFSCEPDRPTSVKGNVESAISSNRMPLMNHFLYTQGALDIETPNVANISTTNSPNTTTVGELGTSLNTCTQQYGRAPTFILVDFFDEGPAINAVDTINGITPTGRTALPARDTTTGRADSSFVGIESLVEQVTEGETPKKAAWIWAAGAWSFGGINLSGGDVVG

**>g7983.t1**

MKLFTKLSLGYSPVGVLLLSALPQTCEGLPQPAHVTSRDTSETGTAQLELPCGPILAIQDDGIYHARGIPYASAQRFEKPSPVNWTAVKDCTQPAPICPQNPSRFDNITGPLTAGRQQSEDCLSVSVAAPVNAQNLPVIVFFHGGAYVTGGGDLAAYSPVPMAQHGAVVVTVTSRLGLLGYLPIPGIAPANLGLFDQIEALRWVQSNIHAFGGNPDSVTIDGQSAGGDAVYCMLAADNTAGLFHHAILESAPLGRLNETSRSAMTAAMSQYAAAKLSASGSATPVPLTHLLALQTAVLGIAAPIDDTLLPYAPVFGAAPLPPLHTASQAVDAAAQHTPVMVGYNANDGYAFGVLLPAHTAAYYQTAVFADGATQLRANISAATGRTPPMYLFEWAPAAADSPWGAVHTLELPFLYGEWSAWADAPMLNGSESRGVVRRVGAQMKELWIAFARGVALQGQVFVIDAGFEFESGGGAGGGGWYAW

**>g8978.t1**

MADVVTSITRLMSRGLQSSMPRLQHFVTCWLASYGVLSSALPTSSYVKLDGYGKFAGTTINSTLQGSPLNDTVEAWLGLDYATQPVGENRFKPVTWPAPFHGVREAKEYGKACVQDSSYVSLDLQDEACLNMNVFRPAGTPKDAQLPTLIWIHGGAFVAGGWMNFDGPAFIASSKVPVMVVTFHYRVNSLGFVPSPLFEEEGLLNLGLRDQHLLLEFVQKHISSFGGDPDAVTLGGRSAGAHSTGIHYFHNYGIDENRPLFARAIHQSGSVTARAFPNATYPLYLNQFDTYMSYLGCPSEDNDAALACLRAANIDDIREISTQLYDNSTDAITWPFQPTQGGPMLEKFGSQSGYDGTFFHVPTITSTVTNEGKYYTRGDLETNQEFLDFMHNTSPELNSTDMELMEQLYPDPATDPTSPYANSPNSTQYDRLSAAWSDYAYICPSQETAYRASSAGVPVWKARFDTNNSYPAWQGIPHTADTRYTWDDTRVQYPEIAHIYHGYLSSFVASGDPNTYRYPGSPVWETYVPGGNDAQHDLAEQLVIHPGATMMETDSIRREACLYWRDPERAPRLNK
